# *Brucella melitensis* UGPase inhibits the activation of NF-κB by modulating the ubiquitination of NEMO

**DOI:** 10.1186/s12917-021-02993-9

**Published:** 2021-08-30

**Authors:** Yucheng Zhou, Zhaoyang Bu, Jing Qian, Yuening Cheng, Lianjiang Qiao, Sen Yang, Shipeng Cheng, Xinglong Wang, Linzhu Ren, Yanling Yang

**Affiliations:** 1 State Key Laboratory for Molecular Biology of Special Economic Animals, Institute of Special Wild Economic Animals and Plants, Chinese Academy of Agricultural Sciences, 130112 Changchun, China; 2 Military Veterinary Institute, Academy of Military Medical Sciences, 130112 Changchun, China; 3 Institute of Veterinary Medicine, Jiangsu Academy of Agricultural Sciences, 210014 Nanjing, China; 4 College of Animal Sciences, Jilin University, 130062 Changchun, China

**Keywords:** *Brucella melitensis*, UTP-glucose-1-phosphate uridylyltransferase (UGPase), Nuclear factor-kappa enhancer-binding protein (NF-κB), Ubiquitination

## Abstract

**Background:**

UTP-glucose-1-phosphoryl transferase (UGPase) catalyzes the synthesis of UDP-glucose, which is essential for generating the glycogen needed for the synthesis of bacterial lipopolysaccharide (LPS) and capsular polysaccharide, which play important roles in bacterial virulence. However, the molecular function of UGPase in *Brucella* is still unknown.

**Results:**

In this study, the ubiquitination modification of host immune-related protein in cells infected with UGPase-deleted or wild-type *Brucella* was analyzed using ubiquitination proteomics technology. The ubiquitination modification level and type of NF-κB Essential Modulator (NEMO or Ikbkg), a molecule necessary for NF-κB signal activation, was evaluated using Coimmunoprecipitation, Western blot, and dual-Luciferase Assay. We found 80 ubiquitin proteins were upregulated and 203 ubiquitin proteins were downregulated in cells infected with *B. melitensis* 16 M compared with those of *B. melitensis* UGPase-deleted strain (16 M-UGPase^−^). Moreover, the ubiquitin-modified proteins were mostly enriched in the categories of regulation of kinase/NF-κB signaling and response to a bacterium, suggesting *Brucella* UGPase inhibits ubiquitin modification of related proteins in the host NF-κB signaling pathway. Further analysis showed that the ubiquitination levels of NEMO K63 (K63-Ub) and Met1 (Met1-Ub) were significantly increased in the 16 M-UGPase^−^-infected cells compared with that of the 16 M-infected cells, further confirming that the ubiquitination levels of NF-κB signaling-related proteins were regulated by the bacterial UGPase. Besides, the expression level of IκBα was decreased, but the level of p-P65 was significantly increased in the 16 M-UGPase^−^-infected cells compared with that of the 16 M- and mock-infected cells, demonstrating that *B. melitensis* UGPase can significantly inhibit the degradation of IκBα and the phosphorylation of p65, and thus suppressing the NF-κB pathway.

**Conclusions:**

The results of this study showed that *Brucella melitensis* UGPase inhibits the activation of NF-κB by modulating the ubiquitination of NEMO, which will provide a new scientific basis for the study of immune mechanisms induced by *Brucella*.

**Supplementary Information:**

The online version contains supplementary material available at 10.1186/s12917-021-02993-9.

## Background

Brucellosis caused by *Brucella spp*. is a widespread zoonosis mainly transmitted from cattle, sheep, goats, pigs, and camels through direct contact with blood, placenta, fetuses, or uterine secretions, or consumption of contaminated animal products [1, 2]. *Brucella* is a typical intracellular parasite, which can establish chronic infection after entering cells, thus avoiding natural immune recognition of the host, resulting in serious public health consequences [3, 4]. However, the specific mechanism is not clear. Therefore, it is an urgent task to screen and identify important virulence factors of *Brucella* and reveal the molecular mechanism of *Brucella* regulating host immune response.

For the first time, we found that UTP-glucose-1-phosphate uridylyltransferase (also known as UDP-glucose pyrophosphorylase, UGPase) has a significantly different expression pattern in *Brucella* vaccine strain M5 and virulent strain 16 M [5], and.

*Brucella* UGPase affects the synthesis, structural integrity and reactivity of lipopolysaccharide (LPS), and the activation of nuclear factor κB (NF-κB), thus affecting the virulence of bacteria, suggesting that UGPase plays an important role in *Brucella* infection and its pathogenicity. However, the mechanism of activation of NF-κB regulated by *Brucella* UGPase is still unknown.

UDP-glucose acts as a glucose-based donor for carbohydrate biosynthesis in prokaryotes, which is an essential substance for the synthesis of LPS, capsular polysaccharides, and membrane-derived oligosaccharides [6–8]. In these biological processes, UGPase, as a key enzyme, promotes UDP-glucose to regulate the structure of bacterial capsules and LPS, thus affecting bacterial virulence [6–8]. Studies have shown that UGPase deletion can lead to changes in the capsular structure of *Streptococcus pneumoniae* [9], affect flagellar formation in *Escherichia coli* [10], reduce biofilm formation of *Pseudomonas syringae* [11], and decrease the virulence of *Klebsiella pneumoniae* [12]. Similarly, the absence of UGPase hinders the corneal infection and systemic spread of *Pseudomonas aeruginosa* [13].

In the present study, the ubiquitination modification of host immune-related protein in cells infected with UGPase-deleted *Brucella* was analyzed using ubiquitination proteomics technology. Moreover, the ubiquitination modification level and type of NF-κB Essential Modulator (NEMO or Ikbkg), a molecule necessary for NF-κB signal activation, was evaluated. This study provides a new theoretical basis for clarifying the molecular mechanism of *Brucella* escaping host immune recognition and inhibiting host immune response.

## Results

### Proteomic profiling of ubiquitination regulated by UGPase of B. melitensis

**Fig. 1:**
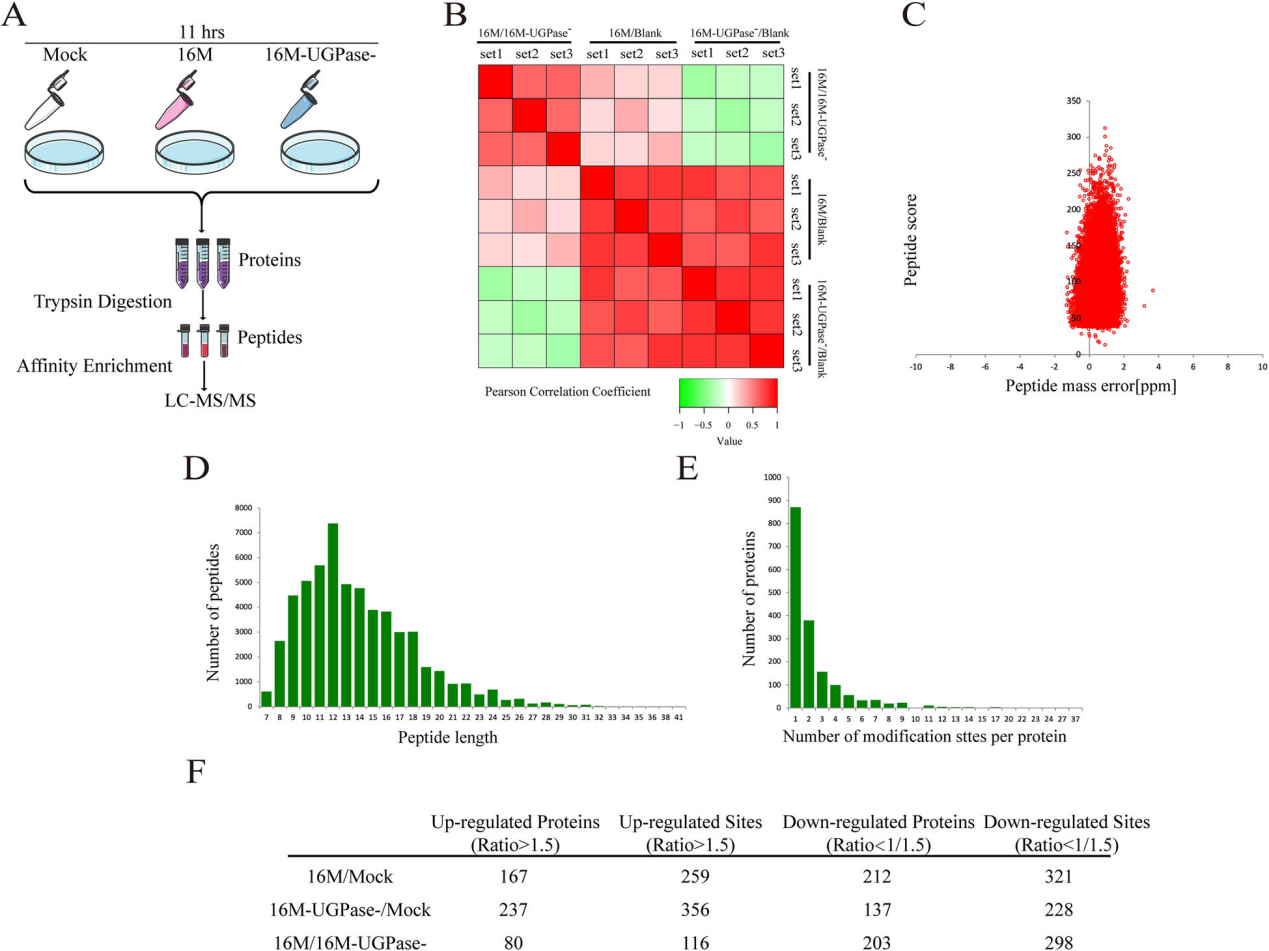
Overview of treatments and proteome profiling. (**A**) Schematic illustration of the experimental procedures used in the present study. Briefly, RAW264.7 cells were subjected to *B. melitensis* 16 M or 16 M-UGPase^−^ infection for 11 h at an MOI of 100. Then, total protein was extracted from the infected cells and digested by trypsin. The tryptic peptides were desalted and enriched by immunoaffinity purification and then analyzed using LC-MS/MS. (**B**) Repetitive analysis. The heat map was generated by calculating Pearson’s correlation coefficient between every sample to measure the degree of linear correlation between the two sets of data. Green indicates a negative correlation, and red indicates a positive correlation. Each group has two replicates and each experiment was repeated three times. (**C**) Distribution of peptides based on mass error. (**D**) Peptide length distribution of all peptides. (**E**) Distribution of peptides based on the number of ubiquitination sites. (**F**) Differentially expressed ubiquitin-modified sites and the corresponding proteins of different treatment groups (*p* < 0.05)

To elucidate the molecular mechanism by which UGPase inhibits the activation of NF-κB, RAW264.7 cells were infected with *B. melitensis* 16 M and 16 M-UGPase^−^, followed by proteomic analysis using ubiquitination enrichment-based label-free quantitative technology and high-resolution LC-MS/MS (Fig. 1 A). The results showed that the quantitative results were highly repetitive and consistent, according to the statistical analyses (Fig. 1B). Data were searched using the SwissProt mouse server and validated and quantified using MaxQuant software (V1.5.2.8). As expected, the mass error for all the protein fragments was near zero, ranging between − 2 and 4 ppm (Fig. 1 C), and most of the peptides were 8–20 amino acids long (Fig. 1D). Furthermore, most of the peptides contained 1–2 ubiquitinated sites (Fig. 1E). These results indicate that the peptides have properties consistent with tryptic peptides. Moreover, 4196 ubiquitinated sites and 1718 ubiquitinated proteins from three groups were identified using ubiquitination enrichment-based label-free quantitative proteomics, and among these identified proteins, 3537 ubiquitinated sites on 1506 Ubiquitinated proteins were quantified (data not shown). The various ubiquitin-modified sites and the differentially expressed corresponding proteins, as determined for different treatment groups, are summarized in Fig. 1 F. As shown in Fig. 1 F, there were 731 ubiquitin-modified sites and 484 ubiquitin proteins with a statistically significant increase in three different combinations, while 847 ubiquitin-modified sites and 552 ubiquitin proteins were significantly decreased. Compared with those in *B. melitensis* 16 M-UGPase^−^, 80 ubiquitin proteins were upregulated, and 203 ubiquitin proteins were downregulated in cells infected with *B. melitensis* 16 M (Fig. 1 F).

### UGPase regulates ubiquitin modification of host proteins

**Fig. 2:**
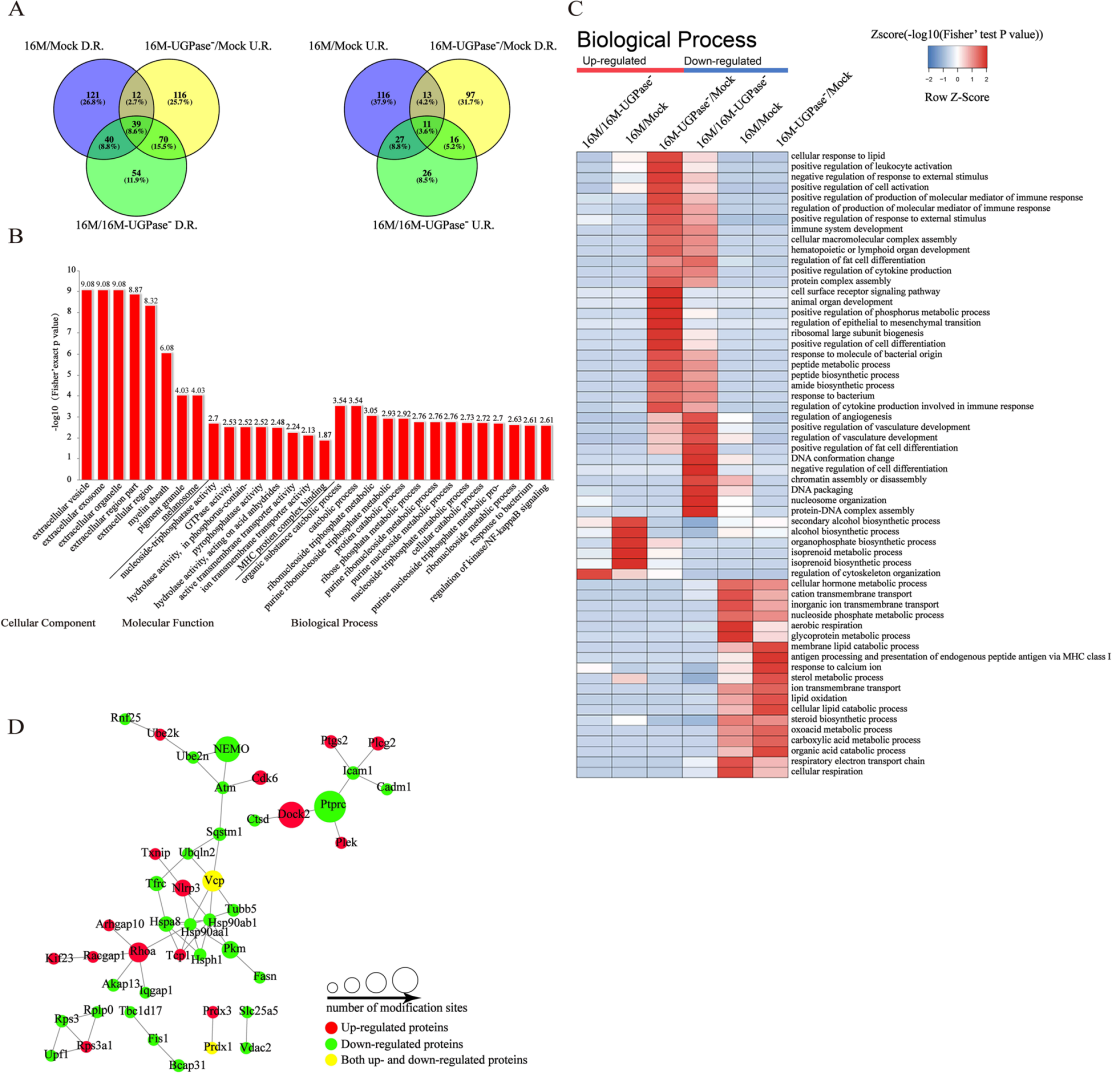
Ubiquitination analysis of host proteins regulated by ***B. melitensis*** UGPase. (**A**) Venn diagram showing a summary of the number of ubiquitinated proteins identified from different combinations. D.R., Downregulated; U.R., Upregulated. (**B**) GO enrichment analysis of the downregulated ubiquitinated proteins. The numbers in the Y axes represent the value as determined from the analysis of significance. Values greater than 1.3 indicate p-values lower than 0.05, which means that the data are statistically significant. (**C**) Heat maps of the biological processes of the identified and quantified proteins. The lowest levels of enrichment are shown in blue, and the highest levels of enrichment are shown in red. (**D**) High-confidence STRING network of proteins with significantly changed ubiquitination levels. The upregulation (16 M/16 M-UGPase^−^) of proteins with ubiquitination sites is marked red, downregulated (16 M/16 M-UGPase^−^) proteins are in green, and proteins that are both upregulated and downregulated are in yellow

To further clarify the regulatory effect of UGPase on the ubiquitin modification of host immune response-related proteins during *Brucella* infection, we performed a comparative analysis of all the differentially expressed ubiquitin-modified proteins that were obtained as described above. A summary of the ubiquitinated proteins identified from different combinations is shown by the Venn diagram (Fig. 2 A). As shown in Fig. 2 A, we detected a total of 212 proteins that were downregulated in the 16 M group compared to those in the mock group, 203 proteins that were downregulated in the 16 M group compared to those in the 16 M-UGPase^−^ group, and 237 proteins that were upregulated in the 16 M-UGPase^−^ group compared to those in the mock group (Fig. 2 A, left panel). Among these ubiquitinated proteins, 39 proteins were common to all three combinations, while 121, 54, and 116 proteins were unique to combinations of the 16 M/Mock group, 16 M/16 M-UGPase^−^ group, and 16 M-UGPase^−^/Mock group, respectively. Similarly, 167 proteins detected in the 16 M group were upregulated compared to those in the mock group, 80 proteins in the 16 M group were upregulated compared to those in the 16 M-UGPase^−^ group, and 137 proteins in the 16 M-UGPase^−^ group were downregulated compared to those in the mock group (Fig. 2 A, right panel). These results indicate that UGPase can regulate the expression of the ubiquitinated protein.

To better understand the ubiquitin-modified proteins that are regulated by *Brucella* UGPase, we performed a Gene Ontology (GO) functional enrichment analysis (Fig. 2B). We found that the ubiquitin-modified proteins were mostly enriched in the biological process category, followed by the cellular component and molecular function categories. In the biological process category, the highly abundant protein species were enriched in the categories of regulation of kinase/NF-κB signaling and response to a bacterium, whereas those with low abundance were enriched in the categories of nucleosome assembly, chromatin assembly, and nucleosome organization. Therefore, we propose that *Brucella* UGPase can significantly inhibit ubiquitin modification of related proteins in the host NF-κB signaling pathway.

To compare the expression patterns of UGPase-regulated proteins in different combinations, a heat map was generated to analyze the biological processes in which the identified UGPase-regulated proteins are involved (Fig. 2 C). The proteins that were significantly upregulated by UGPase were involved in cell surface receptor signaling pathways, immune system development, the positive regulation of cytokine production, and the response to a bacterium (Fig. 2 C).

We then performed bioinformatics analysis using STRING and Cytoscape to analyze the differentially expressed ubiquitin-modified immune-related proteins in the 16 M/16 M-UGPase^−^ group. The interaction networks of the differentially expressed ubiquitinated proteins are shown in Fig. 2D. The ubiquitin modification of several proteins was significantly inhibited in cells infected with *B. melitensis* 16 M compared with those infected with *B. melitensis* 16 M-UGPase^−^ (Fig. 2D). Among these ubiquitinated proteins, NEMO, Sqstm1, Ube2n, and Akap13 are involved in the activation of the NF-κB signaling pathway. Ptprc is related to the differentiation of lymphocytes, while Ubqln2 and Bcap31 participate in the degradation of endoplasmic reticulum-related proteins. Notably, the ubiquitin modification level of NEMO was significantly downregulated in cells infected with *B. melitensis* 16 M compared with those infected with *B. melitensis* 16 M-UGPase^−^ (Fig. 2D). These results demonstrate that UGPase can inhibit the ubiquitin modification level of NEMO.

Taken together, the bioinformatics analysis results described above show that *Brucella* UGPase regulates ubiquitin modification of host proteins.

### B. melitensis UGPase inhibits the activation of NF-κB by modulating the ubiquitination of NEMO

**Fig. 3:**
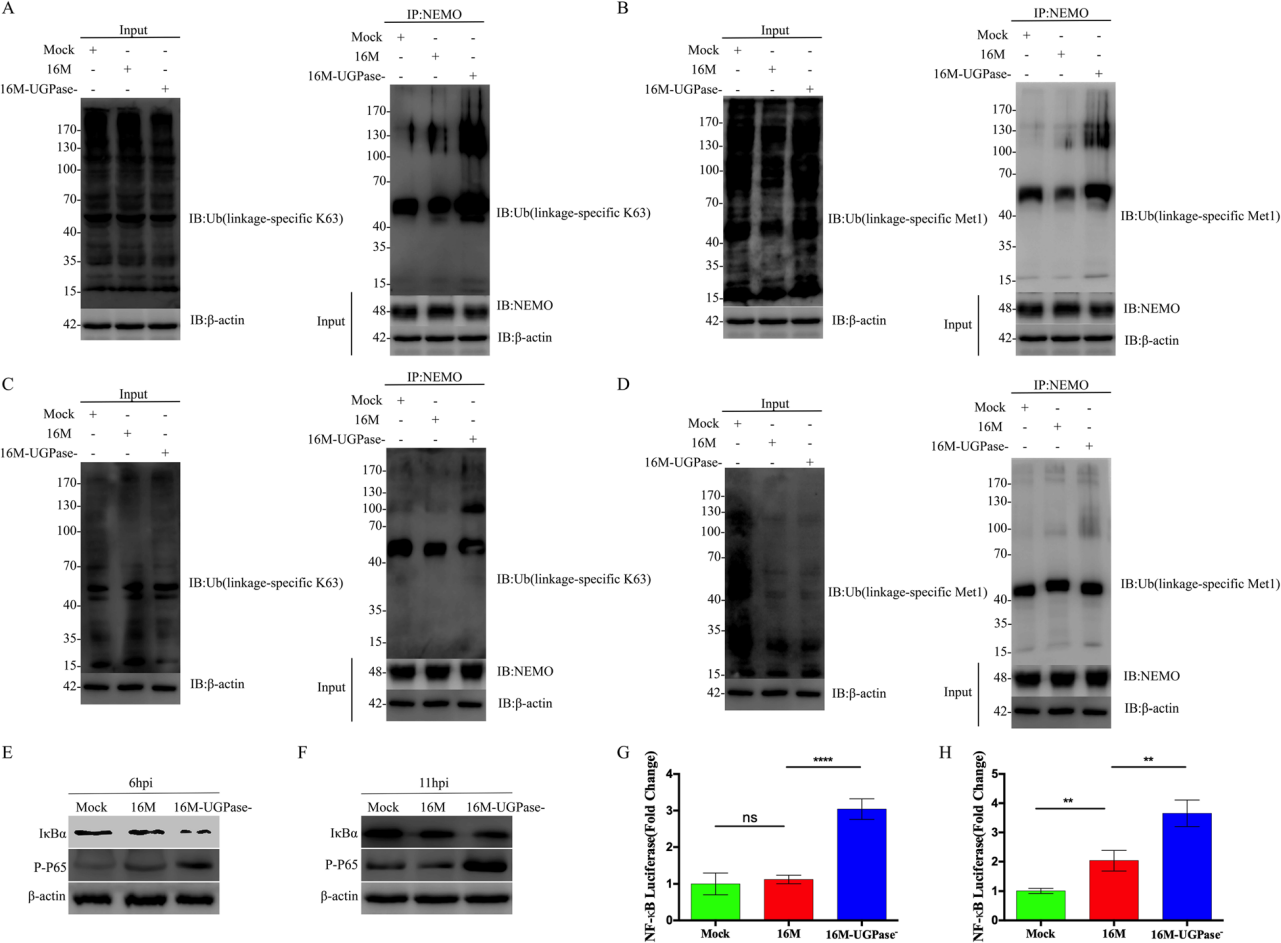
***B. melitensis*** UGPase inhibits the activation of NF-κB by modulating the ubiquitination of NEMO. Cells were infected with 16 M and 16 M-UGPase^−^ for 6 or 11 h. Mock-infected cells were used as negative controls. Whole-cell extracts were collected and divided into two parts. One part was used for coimmunoprecipitation, and the other part was used for immunoblotting analysis. β-actin was used as a negative control for the immunoblotting analysis. (**A**-**B**) Results of Co-IP at 6 hpi. K63-Ub of NEMO (**A**) and Met1-Ub of NEMO (**B**). Left panel, total ubiquitinated K63 or Met1; right panel, ubiquitinated K63 or Met1 of NEMO. (**C**-**D**) Results from the Co-IP at 11 hpi. K63-Ub of NEMO (**C**) and Met1-Ub of NEMO (**D**). Left panel, total ubiquitinated K63 or Met1; right panel, ubiquitinated K63 or Met1 of NEMO. (E-F) Expression levels of IκBα and p-P65 at 6 hpi (**E**) and 11 hpi (**F**). (**G**-**H**) Levels of the regulated NF-κB at 6 (**G**) and 11 hpi (**H**). The activation of the NF-κB signaling pathway was evaluated using a Dual-Luciferase® Reporter 1000 assay system. Unprocessed original scans of the Western blots can be found in Supplementary Fig. S1, S2, S3, S4, S5, S6

NEMO is an essential regulator of the NF-κB signaling pathway, and ubiquitination of the K63 and Met1 sites of NEMO is stimulated by IL-1β and TNF-α, respectively [14, 15]. Ubiquitinated NEMO can produce cascade signals that lead to the degradation of downstream IκBα by the proteasome and the release of the NF-κB dimer [16]. Thereafter, P65 (RelA) is activated by phosphorylation and transferred into the nucleus to activate the transcription of inflammatory cytokines [16]. In this study, cells were infected with *B. melitensis* 16 M and 16 M-UGPase^−^, and the ubiquitination levels of K63 (K63-Ub) and Met1 (Met1-Ub) were analyzed at 6 and 11 h postinfection (hpi). The results showed that the expression levels of total ubiquitinated K63 (K63-Ub) in the host cell were similar in the 16 M-, 16 M-UGPase^−^- and mock-infected cells (Fig. 3 A, left panel), but the ubiquitinated K63 of NEMO (K63-Ub) was significantly increased in the 16 M-UGPase^−^-infected cells compared with the ubiquitination of K63 in the 16 M- or mock-infected cells at 6 hpi (Fig. 3 A, right panel). The NEMO K63-Ub level in the 16 M-infected cells was also slightly greater compared with that of the mock-infected group at 6 hpi (Fig. 3 A, right panel). Furthermore, NEMO Met1-Ub was also significantly greater in the 16 M-UGPase^−^ -infected cells at 6 hpi, while NEMO Met1-Ub was significantly decreased in the 16 M-infected cells at 6 hpi compared with Met1-Ub in the mock-infected group (Fig. 3B, right panel). At 11 hpi, the total K63-Ub was similar in two infection groups (Fig. 3 C, left panel), while the NEMO K63-Ub level in the 16 M-infected cells was lower compared with that in the mock-infected group (Fig. 3 C, right panel). Interestingly, the levels of the total Met1-Ub chain in the host cell were significantly decreased in the 16 M- and 16 M-UGPase^−^-infected cells compared with that the level in the mock-infected group (Fig. 3D, left panel), suggesting *Brucella* can inhibit the formation of Met1 ubiquitin chain by the bacterial UGPase. Meanwhile, NEMO Met1-Ub in the 16 M-UGPase^−^-infected cells was similar to the level in the 16 M-or mock-infected cells at 11 hpi (Fig. 3D, right panel). These results suggested that the ubiquitination levels of NEMO K63 (K63-Ub) and Met1 (Met1-Ub) were regulated by the bacterial UGPase during the infection.

To further analyze the effect of *B. melitensis* UGPase on the activation of NF-κB, the expression levels of IκBα and the level of phosphorylated P65 (p-P65) were detected in the 16 M-, 16 M-UGPase^−^- and mock-infected cells. The results demonstrated that the expression level of IκBα was decreased, but the level of p-P65 was significantly increased in the 16 M-UGPase^−^-infected cells compared with the expression and phosphorylation in the 16 M- and mock-infected cells at 6 and 11 hpi (Fig. 3E and F). These results were further confirmed by the dual-luciferase reporter assay. As shown in Fig. 3G and H, the levels of luciferase, as regulated by the NF-κB response element, were significantly increased in the 16 M-UGPase^−^-infected cells compared with the level in the 16 M- and mock-infected cells at 6 hpi and 11 hpi (Fig. 3G and H). These results indicated that *B. melitensis* UGPase can significantly inhibit the degradation of IκBα and the phosphorylation of p65, and thus suppressing the NF-κB pathway.

Therefore, these results demonstrate that *B. melitensis* UGPase inhibits the activation of NF-κB by modulating the ubiquitination of NEMO.

## Discussion

Ubiquitination is the most common posttranslational modification of proteins in eukaryotes and controls cell cycle progression, transcriptional regulation, signal transduction, immune response, and so on [17]. During infection, many pathogens establish complex interactions between their virulence factors and the defense mechanisms of the host; however, the ubiquitination system, an important component of cell defense, can regulate the signal transduction of pattern recognition receptors, mediate the innate immune response, and initiate the adaptive immune response [17, 18]. It was reported that the *Yersinia* virulence factor YopJ acts as a deubiquitinase that inhibits NF-κB activation [19], and the *Shigella flexneri* effector OspG interferes with innate immune responses by targeting ubiquitin-conjugating enzymes [20]. However, the molecular mechanism of *Brucella* and related virulence factors in affecting host ubiquitin modification and the immune response is still unclear. In this study, we analyzed the ubiquitin modification of cells infected with a mutant strain and the wild-type parent strain and then screened for and identified the immune-related proteins that were obviously different in the mutant-infected cells and the parent strain-infected cells, especially ubiquitin modification proteins related to NF-κB activation. As shown in Fig. 2, we found that UGPase can significantly regulate the ubiquitin modification of host immune-related proteins during *Brucella* infection, with most of these proteins involved in antigen presentation, NF-κB signal activation, lymphocyte proliferation, cytokine secretion, the inflammatory response, endoplasmic reticulum-related protein degradation, lipid depletion, and polarization. For example, intercellular adhesion molecule 1 (ICAM-1) facilitates antigen presentation of target cells [21], and cell adhesion molecule-1 (CADM1) promotes natural killer cell cytotoxicity, interferon secretion, apoptosis, and oncogenesis [22, 23]. Furthermore, NEMO [24], ubiquitin-binding protein p62 (also named SQSTM1) [25], ubiquitin-conjugating enzyme E2 N (Ube2n) [26], and A-kinase anchoring protein (Akap13) [27] are involved in the activation of the NF-κB signal pathway. In addition, protein tyrosine phosphatase receptor type C (Ptprc) is related to the differentiation of lymphocytes [28], and ubiquilin 2 (Ubqln2) and B cell receptor associated protein 31 (Bcap31) participate in the degradation of the endoplasmic reticulum-related proteins [29, 30]. These results indicate that ubiquitin modification plays an important regulatory role in the host immune response during *Brucella* infection, and these findings are the first to be published based on *Brucella* research. This study opens a new research field and provides a new scientific basis for discovering the immune mechanism of *Brucella*. However, the molecular mechanism by which these ubiquitination proteins regulate the immune response during *Brucella* infection still requires further investigation.

Cellular TLR4 recognizes bacterial LPS during a *Brucella* infection and recruits the IRAK1/4 complex through the adaptor protein MyD88, and then, the activated IRAK1/4 complex recruits and activates TRAF6 [31]. In turn, the activated TRAF6 induces the assembly of Ube2n and UEV1A, and the generation of K63-Ub chains [14, 31]. On the other hand, the activated TLR4 also recruits the adaptors TRAM and TRIF [32], the pathways of which activate NF-κB and interferon production through the ubiquitination of NEMO K63-Ub and Met1-Ub [14, 15, 33]. In this study, we also found that *Brucella* UGPase can inhibit the ubiquitination of NEMO, enabling the activation of IκBα and the inhibition of NF-κB, as indicated by the level of IκBα decreasing and by the level of phosphorylated p65 increasing in the UGPase-mutant strain compared with these levels in the parent strain (Fig. 3). When UGPase was deleted, IκBα was targeted for proteasome degradation, and p65 (RelA) was phosphorylated and transferred into the nucleus to regulate the transcription of inflammatory factors, resulting in a significant increase in NF-κB and interferon production. Therefore, these results demonstrate that *B. melitensis* UGPase inhibits the activation of NF-κB by modulating the ubiquitination levels of K63 (K63-Ub) and Met1 (Met1-Ub) of NEMO, and thus avoiding the host immune response and enhancing the survival in cells, which may result in the intracellular survival and replication of *B. melitensis* and the establishment of chronic infection. However, the exact mechanism involved needs to be further investigated.

## Conclusions

In conclusion, our results demonstrate, for the first time, that *Brucella* UGPase inhibits the activation of host NF-κB signaling by regulating the ubiquitination of NEMO. Our findings provide new insights into the mechanism used by *Brucella* to escape the immune recognition by the host and inhibit the immune response of the host.

## Methods

### Bacterial strains and growth media

*B. melitensis* mutant strain (16 M-UGPase^−^), a UGPase gene deleted strain, was constructed previously by deleting the UGPase gene of *B. melitensis* virulent strain 16 M via homologous recombination [34]. TBS agar plates (Fluka, USA) were used to cultured *B. melitensis* virulent strain (16 M) and mutant strain (16 M-UGPase^−^) at 37 ℃.

### ***In vitro*** infection

Murine RAW264.7 macrophages (RAW264.7) were culture as described previously [35] to 80 % confluence. Then, the cells were infected with *B. melitensis* 16 M or 16 M-UGPase^−^ at a multiplicity of infection (MOI) of 100 CFUs per cell for 5 h, and washed with PBS three times, followed by culturing with fresh DMEM (containing 100 µg/mL gentamicin) for indicated hours. The cells were washed with PBS three times and stored for the following studies.

### Protein purification for proteomic analysis

The infected cells obtained above were lysed with 2 mL IP lysis buffer (containing PMSF). The whole-cell proteins were quantified, purified, and digested according to the protocol described previously [35].

The digested liquid was dissolved in IP buffer (Beyotime Biotechnology, Shanghai, China) and incubated with ubiquitin coupled resin (PTM-1 104, PTM Bio, Hangzhou, China) at 4 °C overnight with shaking. The samples were washed with IP buffer four times and then rinsed twice with deionized water before three elutions of 0.1 % trifluoroacetic acid (TFA, Sigma, USA). The flow-through liquid was desalted using C18 ZipTips (Millipore Corp., Billerica, MA, USA) and concentrated by vacuum centrifugation. The amount of protein was quantified with a BCA protein assay kit (Beyotime Biotechnology, Shanghai, China) and analyzed via LC-MS/MS.

### LC-MS/MS and Bioinformatics Analysis

Proteomics analysis was performed by PTM Bio (Hangzhou, China) using a Thermo Scientific EASY-nLC 1000 system (Thermo Scientific, USA) and an OrbitrapFusion™ mass spectrometer (Thermo Scientific, USA). The positive ion mode was used, and data were acquired using the data-dependent acquisition (DDA) method, which dynamically selects 20 of the most abundant precursor ions from a survey scan for higher-energy collisional dissociation (HCD) fragmentation. For the MS analysis, the following parameters were set: the automatic gain control (AGC) target was set to 1E4, the maximum injection time was 100 ms, the dynamic exclusion duration was 15 s, and the signal threshold was set to 5000 ions/s. The raw LC-MS/MS files were analyzed by MaxQuant version 1.5.2.8. The LC-MS/MS spectra were searched in the SwissProt mouse proteome database (16,839 sequences) using the MASCOT search engine 2.3 (Matrix Science) through the Proteome Discoverer (version 1.4.1.14) (Thermo). The identification of all peptides was filtered at a 1 % spectral FDR (false discovery rate). The heat map was generated by calculating the Pearson’s correlation coefficient between every sample to measure the degree of linear correlation between the two sets of data.

### Coimmunoprecipitation (Co-IP)

The infected RAW264.7cells obtained above were lysed with cell lysis buffer in preparation for Western blotting and IP (Beyotime Biotechnology, Shanghai, China). Five hundred microliters of whole-cell extracts were incubated with 1 µg normal mouse IgG (Beyotime Biotechnology, Shanghai, China) and 20 µL of protein A + G agarose (Beyotime Biotechnology, Shanghai, China) at 4 °C for 2 h. After centrifugation for 5 min at 1000 g and 4 °C, the supernatants of the whole-cell extracts were collected and divided into two parts. One part was used for coimmunoprecipitation, and the other part was used for the immunoblotting analysis.

For coimmunoprecipitation, the supernatant of the whole-cell extracts was incubated with 2 µg anti-NEMO antibody (1:10, Abcam, UK) at 4 °C overnight on a rocking platform. Then, the supernatant was incubated with 20 µL protein A + G agarose at 4 °C for 2 h on a rocking platform and centrifuged at 1000 g for 5 min, and then, the precipitate was washed five times with 1 mL of cell lysis buffer for Western blotting and IP.

The precipitate was resuspended in 40 µL of SDS-PAGE loading buffer (1×), centrifuged at high speed for a short time, and boiled for 10 min. The samples were analyzed by Western blotting using an anti-ubiquitin (linkage-specific K63) antibody (1:1000, Abcam, UK) or anti-ubiquitin (linkage-specific Met1) antibody (1:2000, Thermo Fisher, USA) as primary antibodies. The PVDF membranes (Millipore, USA) were then incubated with EasyBlot anti-Rabbit IgG (1:1000, Genetex, California, USA) as a secondary antibody and developed using a BeyoECL Star Western blot detection system (Beyotime Biotechnology, Shanghai, China) according to the manufacturer’s instructions. The whole-cell lysates (input samples) were loaded as the input controls for the Western blot analysis.

### Western blot (WB) analysis

Western blot was performed according to the protocol described previously with a little revision [35]. Briefly, the whole-cell lysates were separated by polyacrylamide gel electrophoresis and electro-transferred onto PVDF membranes, followed by Western blot assay. The anti-NEMO antibody (1:2000, Abcam, UK), anti-ubiquitin (linkage-specific K63) antibody (1:1000, Abcam, UK), anti-ubiquitin (linkage-specific Met1) antibody (1:1000, Thermo Fisher, USA), anti-IκBα antibody (Beyotime Biotechnology, Shanghai, China), anti-p-P65 antibody (Beyotime Biotechnology, Shanghai, China), or anti-β-actin antibody (1:2000, Abcam, UK) was used as primary antibody. HRP-labeled goat anti-rabbit IgG (H + L) (1:1000) was used as the secondary antibody. The results were examined via the BeyoECL Star Western blot detection system.

### Dual-Luciferase Assay

Dual-luciferase assay was performed according to the protocol described previously with a little revision [35]. Briefly, The RAW264.7 cells were cultured in 96-well plates to 70 confluence and co-transfected with 100 ng pGL4.32[luc2P/NF-κB-RE/Hygro] vector and 50 ng pRL-SV40-N vector. Then, the cells were infected with the 16 M or 16 M-UGPase^−^ (MOI = 100) for 5 h, and washed with PBS thrice, followed by culturing with fresh DMEM (containing 100 µg/mL gentamicin) for 1 and 6 h. Thereafter, the activation of the NF-κB signaling pathway was examined by a Dual-Luciferase® Reporter 1000 assay system (Promega, USA). PBS was used as a mock control.

### Statistical Methods

Data were expressed as the mean ± standard deviation (SD). All statistical analyses were conducted using GraphPad Prism 6 (Invitrogen, USA), followed by Student’s t-test to compare the statistical significance. *P* < 0.05 has statistically significant. All experiments were performed three times.

## Supplementary Information

**Additional file 1:**

## Data Availability

All data generated or analysed during this study are included in this published article and its supplementary information files.

## Abbreviations

AGC: Automatic gain control
Co-IP: Coimmunoprecipitation
DDA: Data-dependent acquisition
DTT: Dithiothreitol
DMEM: Dulbecco's minimum essential medium
GO: Gene Ontology
HCD: Higher-energy collisional dissociation
LPS: Lipopolysaccharide
LC-MS/MS: Liquid chromatography mass spectrometry
MOI: Multiplicity of infection
NEMO: NF-κB Essential Modulator
NF-κB: Nuclear factor-kappa enhancer-binding protein
SDS-PAGE: Sodium dodecyl sulfate polyacrylamide gel electropheresis
TLR: Toll-like receptor
TFA: Trifluoroacetic acid
UGPase: UTP-glucose-1-phosphoryl transferase
WB: Western blot
