# Supplementary material for: Brucella melitensis UGPase inhibits the activation of NF-κB by modulating the ubiquitination of NEMO
Source: BMC Vet Res. 2021 Aug 30;17:289. doi: 10.1186/s12917-021-02993-9 (PMC8404259; doi:10.1186/s12917-021-02993-9)
Supplement: Supplementary file 1 — Additional file 1: [file 12917_2021_2993_MOESM1_ESM.docx]

*Brucella melitensis* UGPase inhibits the activation of NF-κB by modulating the ubiquitination of NEMO

**Yucheng Zhou^1*^, Zhaoyang Bu^3*^, Jing Qian^4*^, Yuening Chen^1^, Lianjiang Qiao^1^, Sen Yang^1^, Shipeng Chen^1^, Xinglong Wang^3^, Linzhu Ren^2🖂^, Yanling Yang^1🖂^**

^1^ State Key Laboratory for Molecular Biology of Special Economic Animals, Institute of Special Wild Economic Animals and Plants, Chinese Academy of Agricultural Sciences, Changchun 130112, China

^2^ Jilin Provincial Key Laboratory of Animal Embryo Engineering, College of Animal Sciences, Jilin University, Changchun 130062, China

^3^ Military Veterinary Institute, Academy of Military Medical Sciences, Changchun 130112, China

^4^ Institute of Veterinary Medicine, Jiangsu Academy of Agricultural Sciences, Nanjing 210014, China

Running Title: *B. melitensis* UGPase inhibits NF-κB by ubiquitination

^🖂^Corresponding author:

Yanling Yang ([m18043213639@163.com](mailto:m18043213639@163.com)) and Linzhu Ren ([renlz@jlu.edu.cn](mailto:renlz@jlu.edu.cn))

* These authors contributed equally to this work.

This file includes:

Supplementary Figures: Fig S1 to S6

**Supplementary Figures**

**Fig S-1: Original Western images used for preparing Figure 3A.**

| Input-K63Ub（Lane 1-2-3） | IP:NEMO-K63Ub（Lane 5-6-7） |
| --- | --- |
| 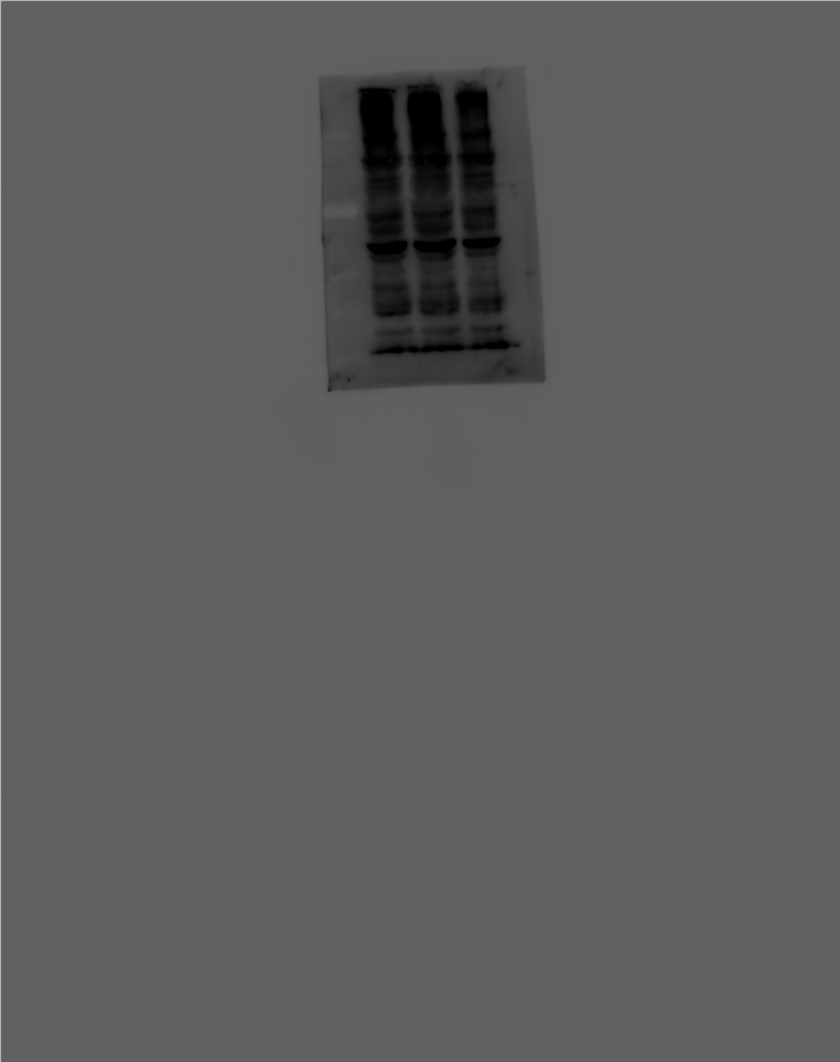 | 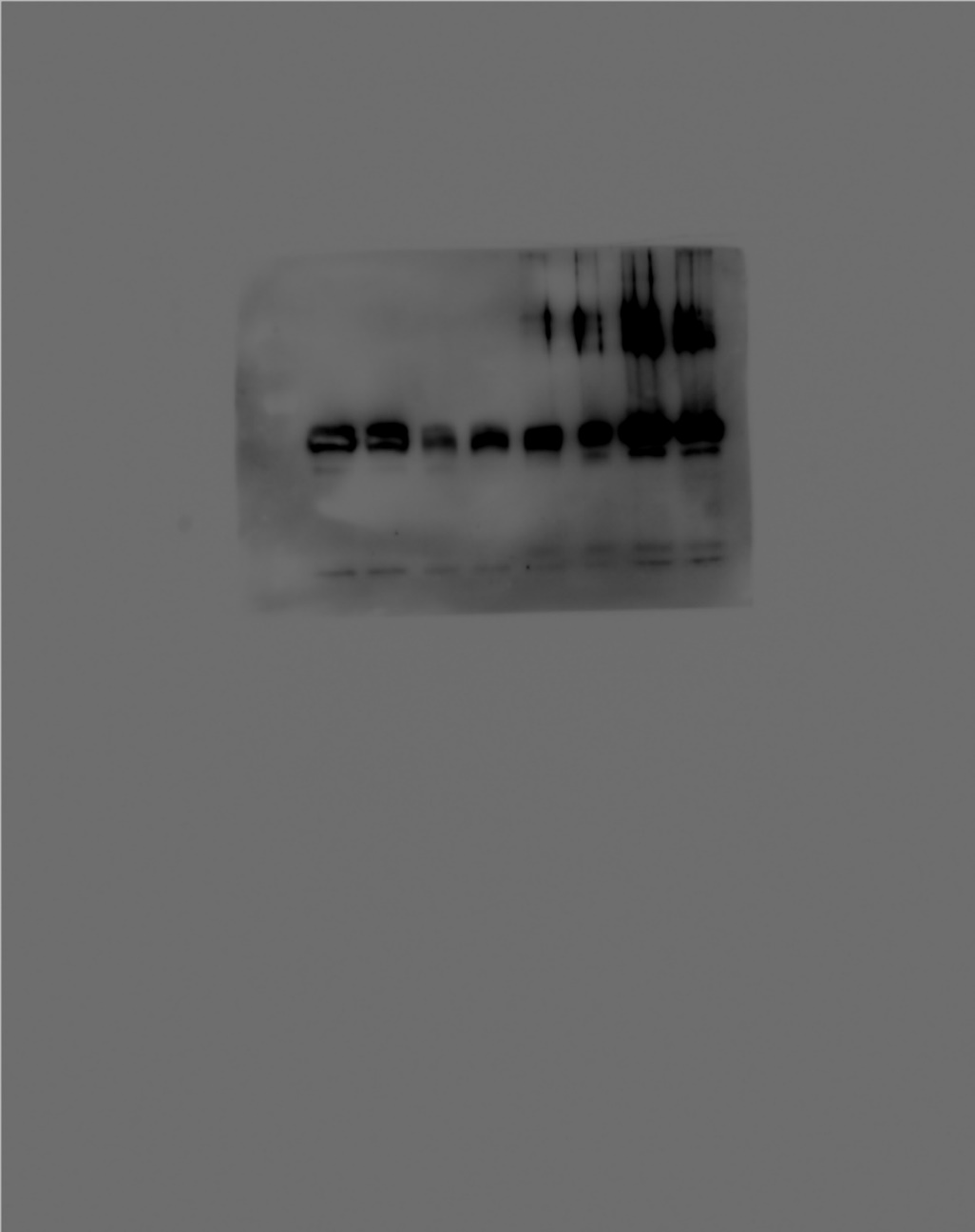 |
| β-actin（Lane 1-2-3） | NEMO（Lane 5-6-7） |
| 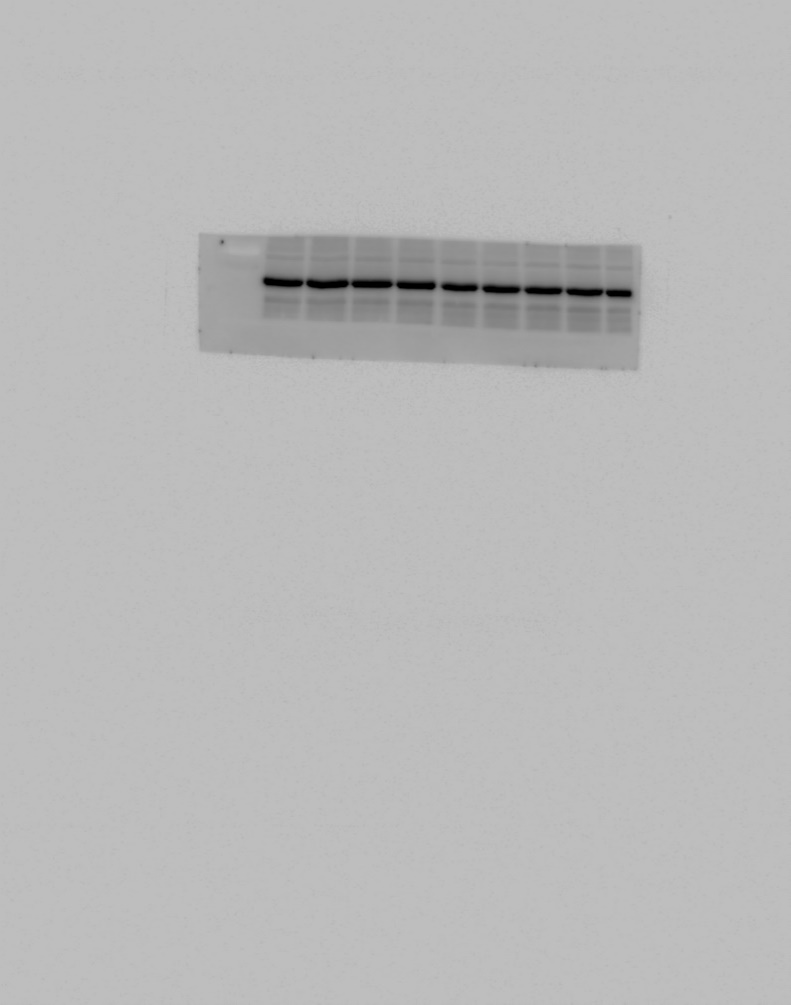 | 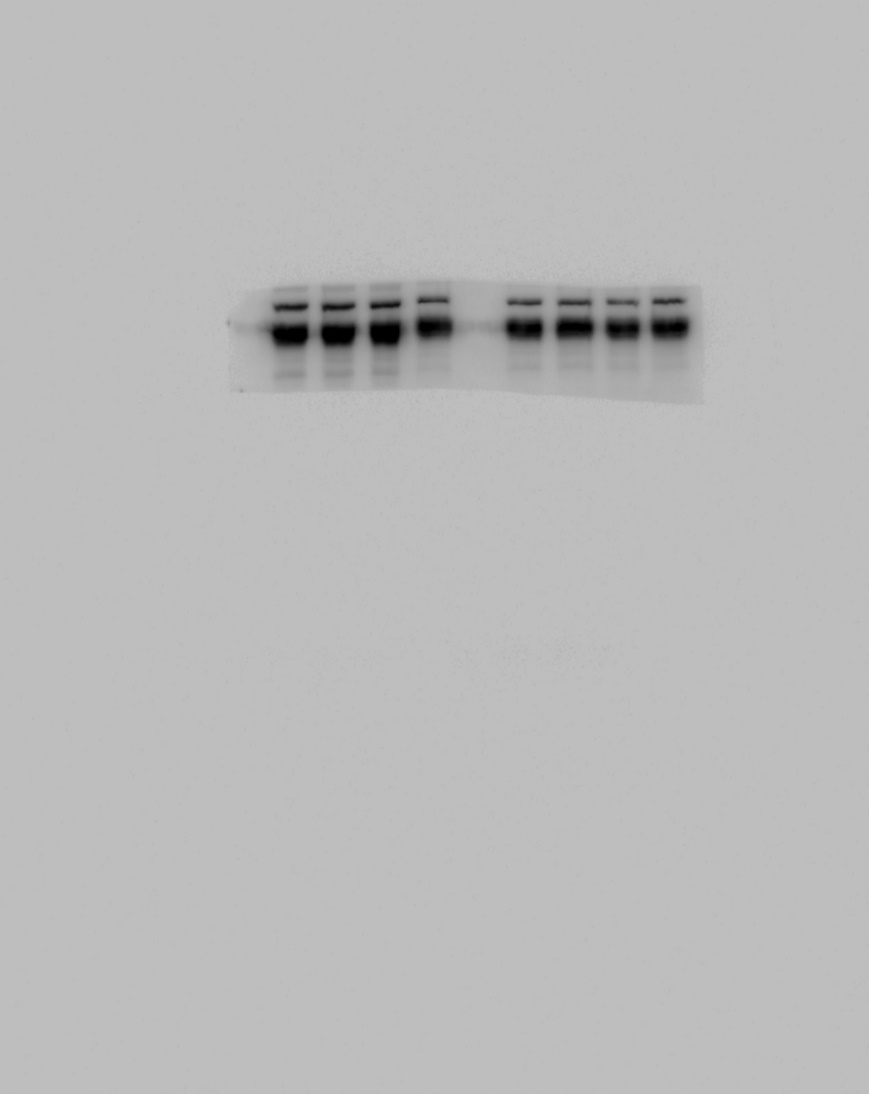 |

**Fig S-2: Original Western images used for preparing Figure 3B.**

| Input-Met1Ub（Lane 1-2-3） | IP:NEMO-Met1Ub（Lane 1-2-3） |
| --- | --- |
| 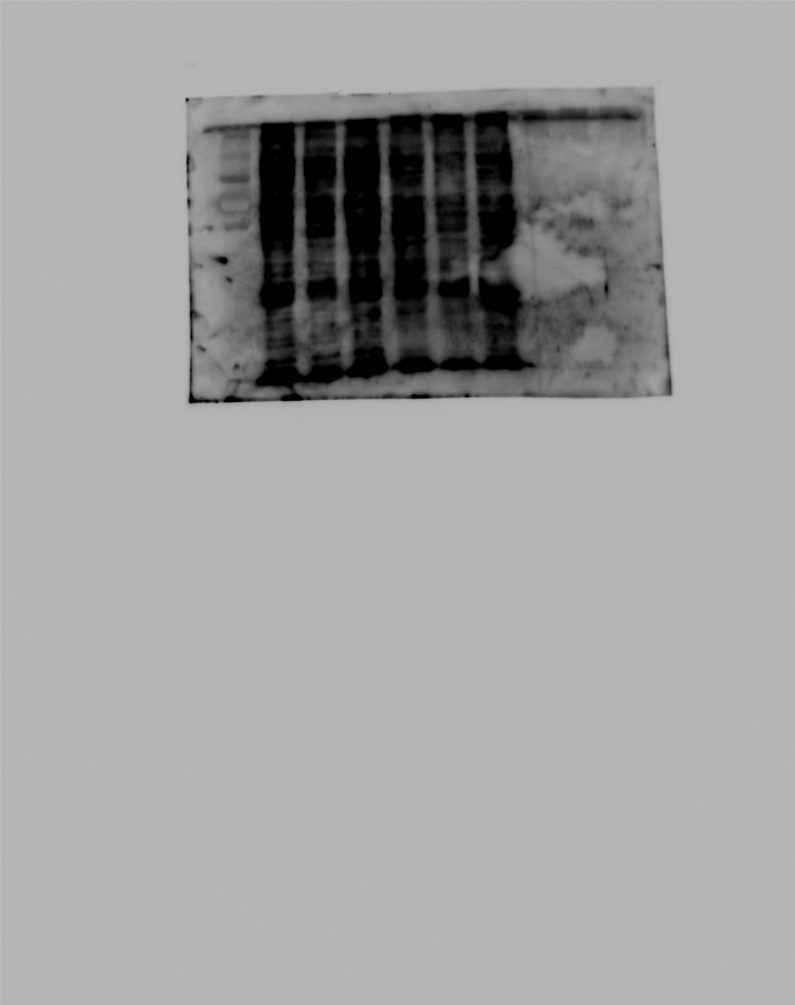 | 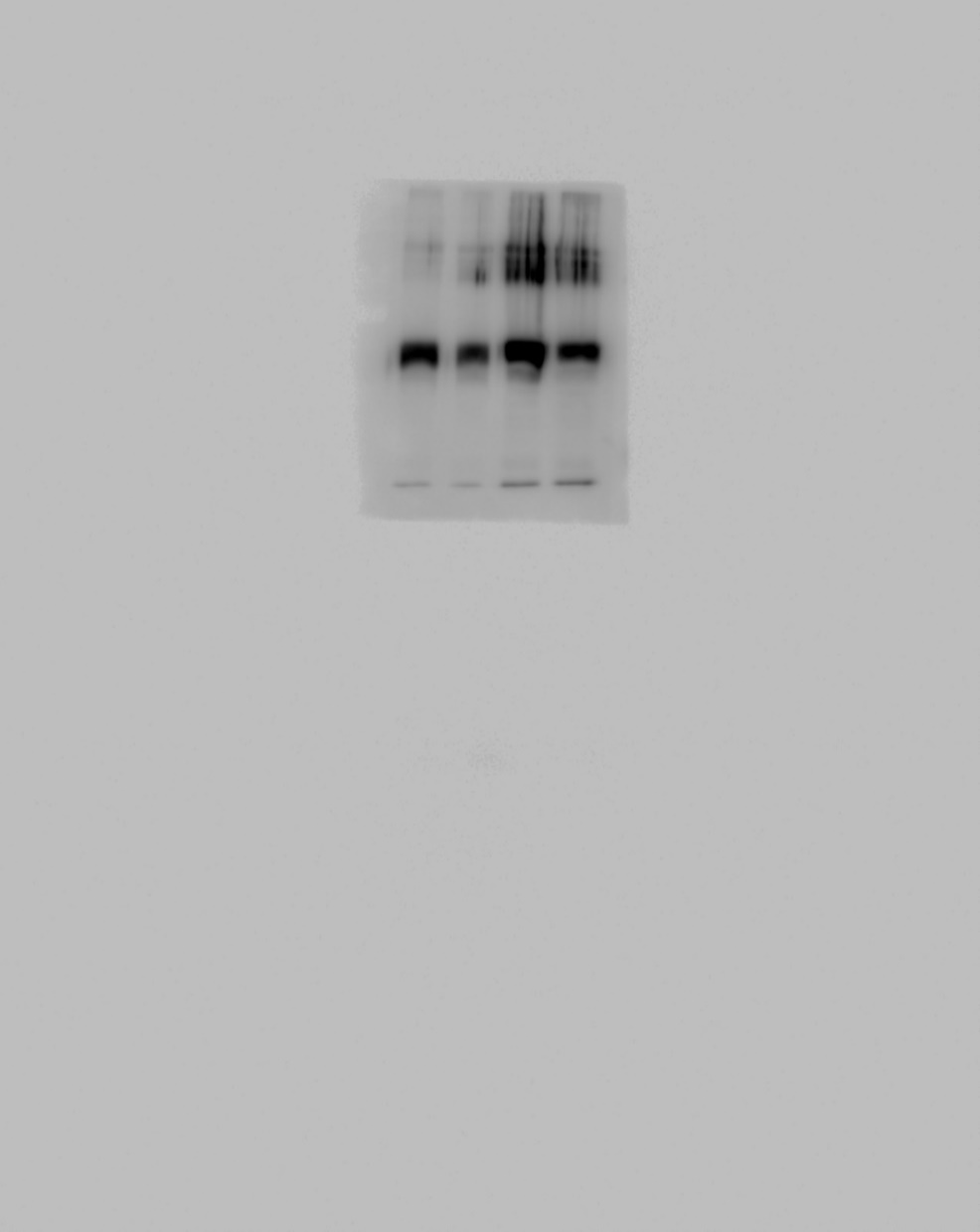 |
| β-actin（Lane 1-2-3） | NEMO（Lane 5-6-7） |
| 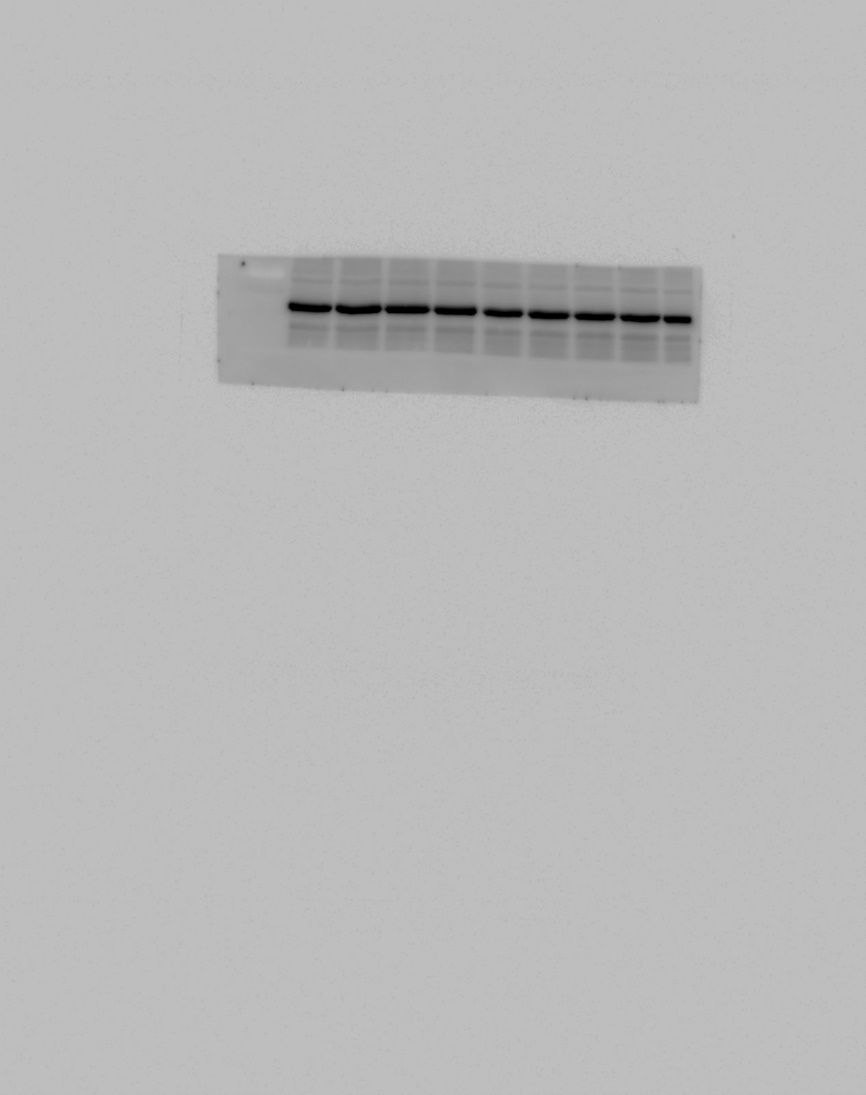 | 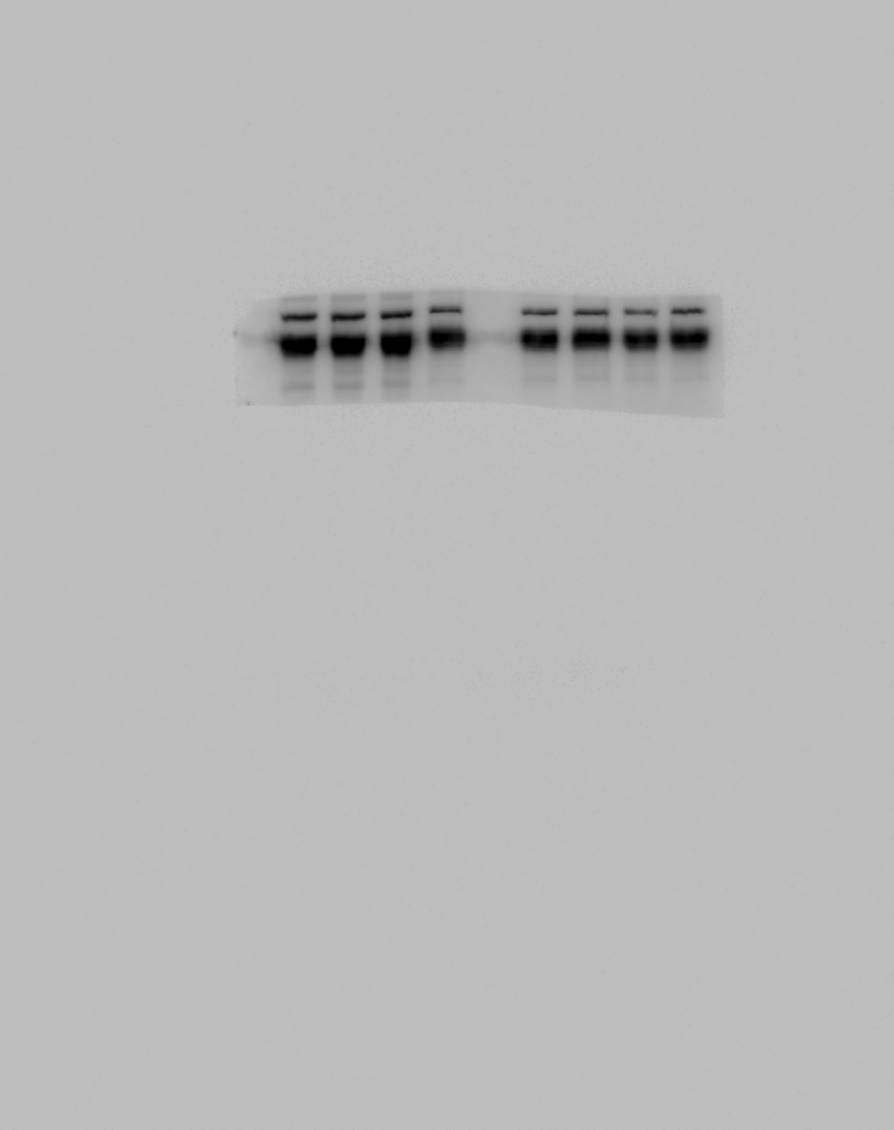 |

**Fig S-3: Original Western images used for preparing Figure 3C.**

| Input-K63Ub（Lane 1-2-3） | IP:NEMO-K63Ub（Lane 1-2-3） |
| --- | --- |
| 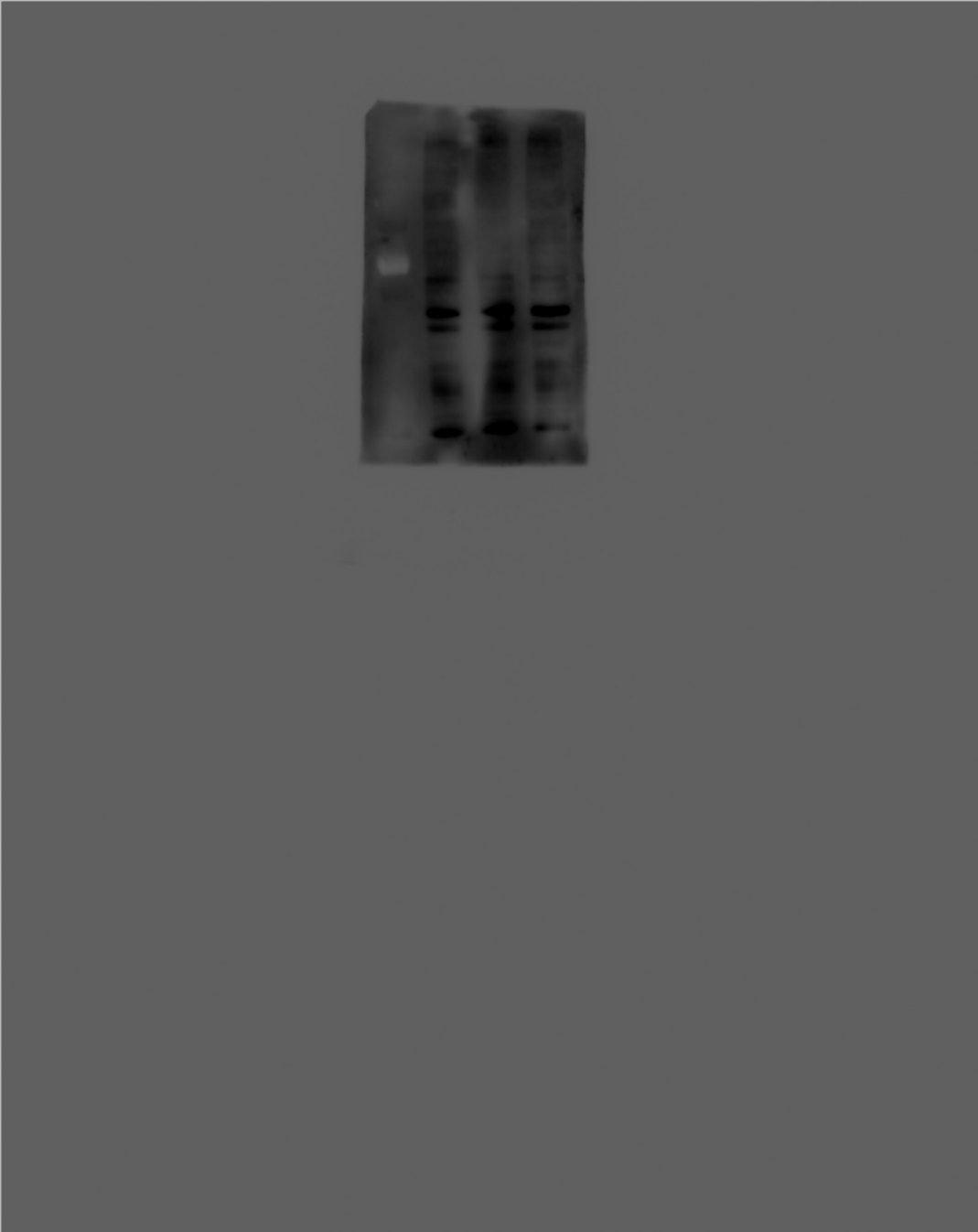 | 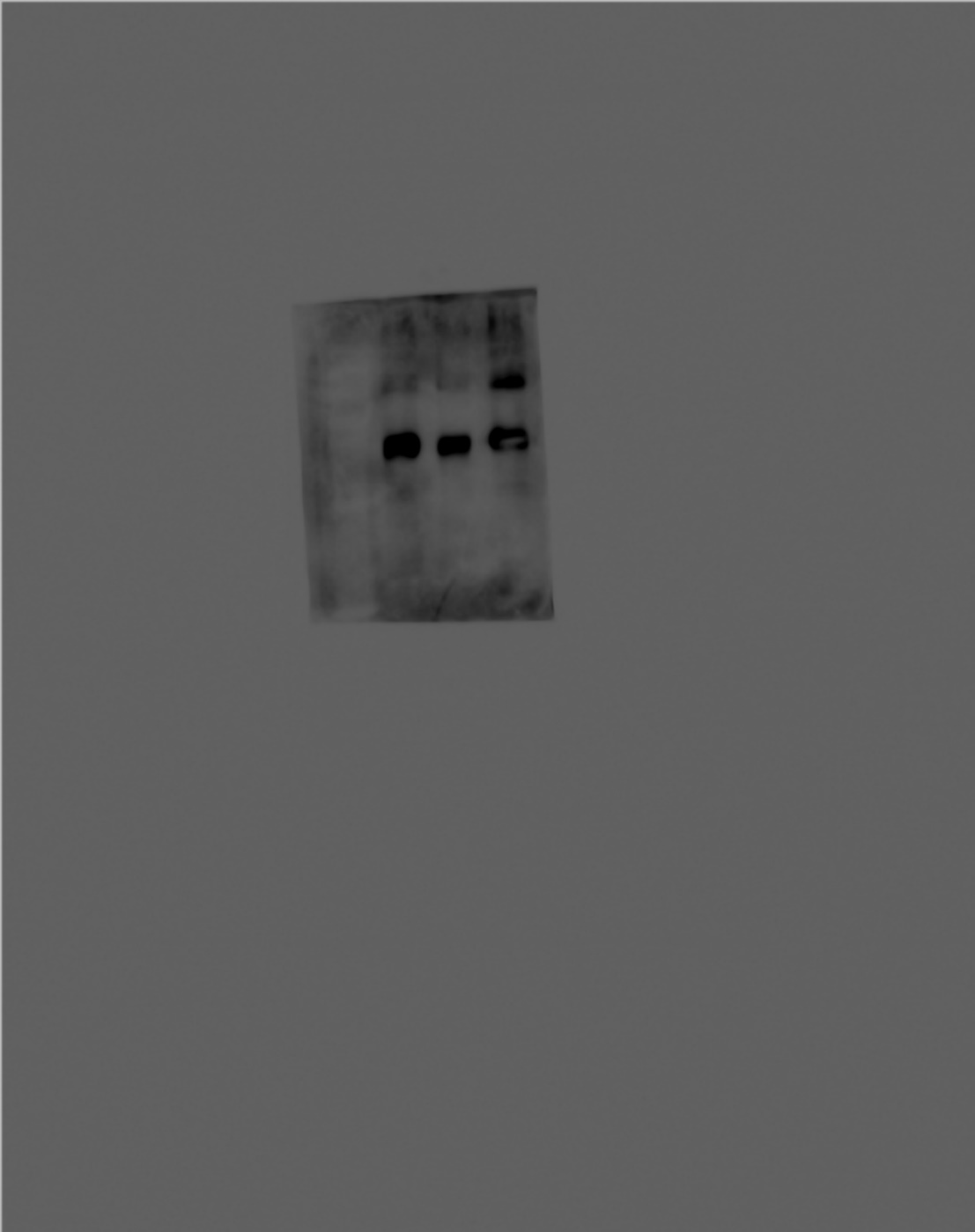 |
| β-actin（Lane 4-5-6） | NEMO（Lane 1-2-3） |
| 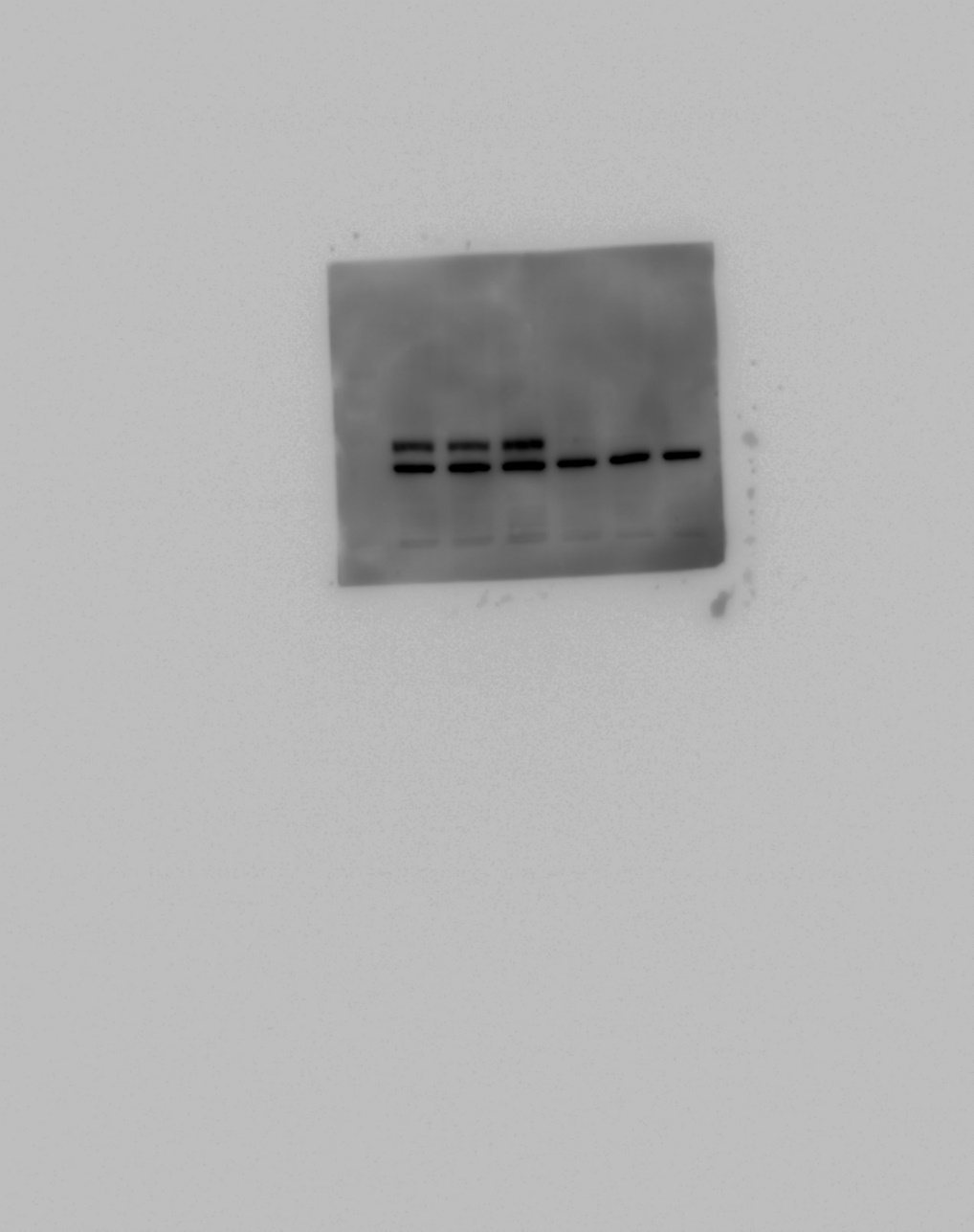 | 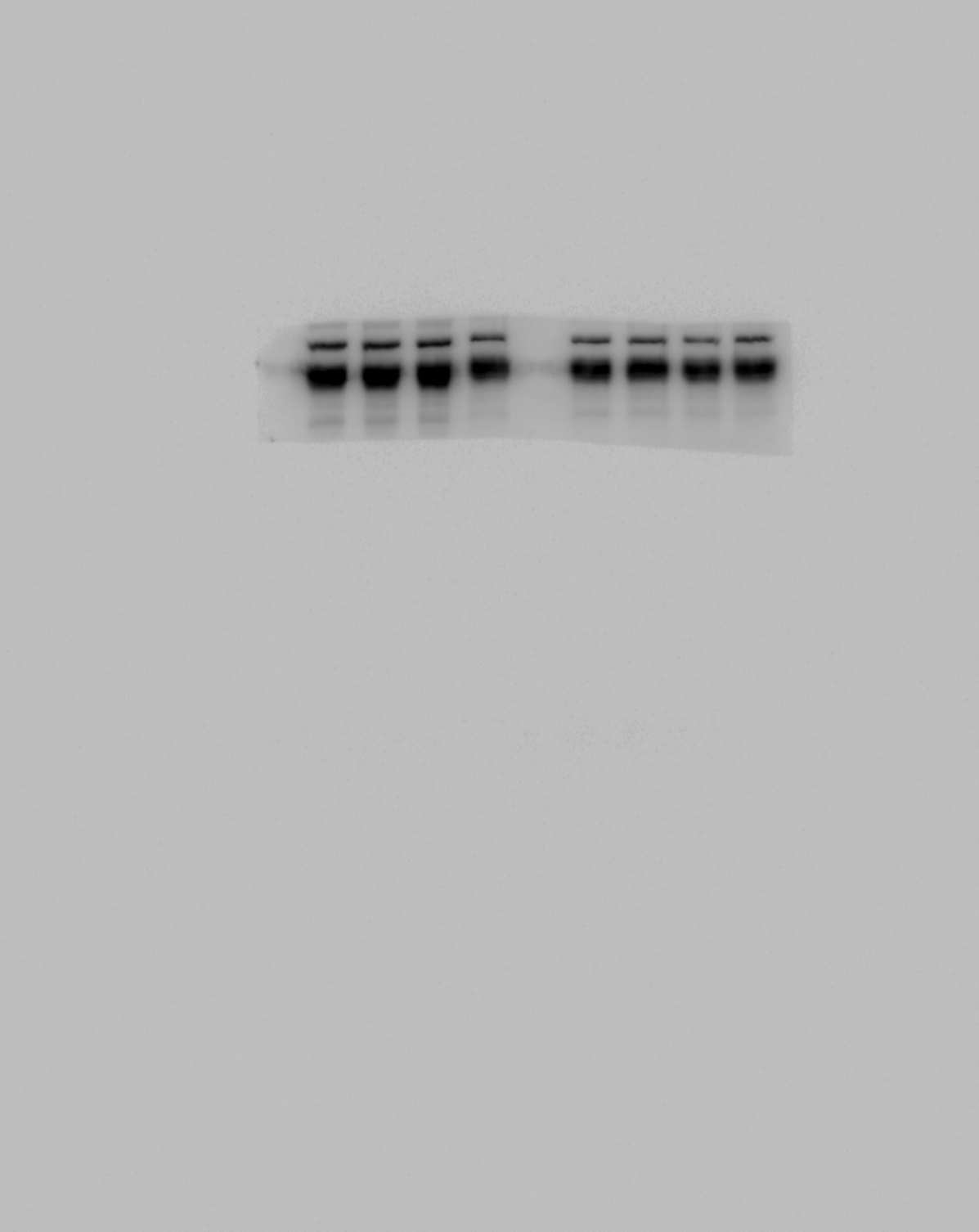 |

**Fig S-4: Original Western images used for preparing Figure 3D.**

| Input-Met1（Lane 2-3-4） | IP:NEMO-ibMet1（Lane 1-2-3） |
| --- | --- |
| 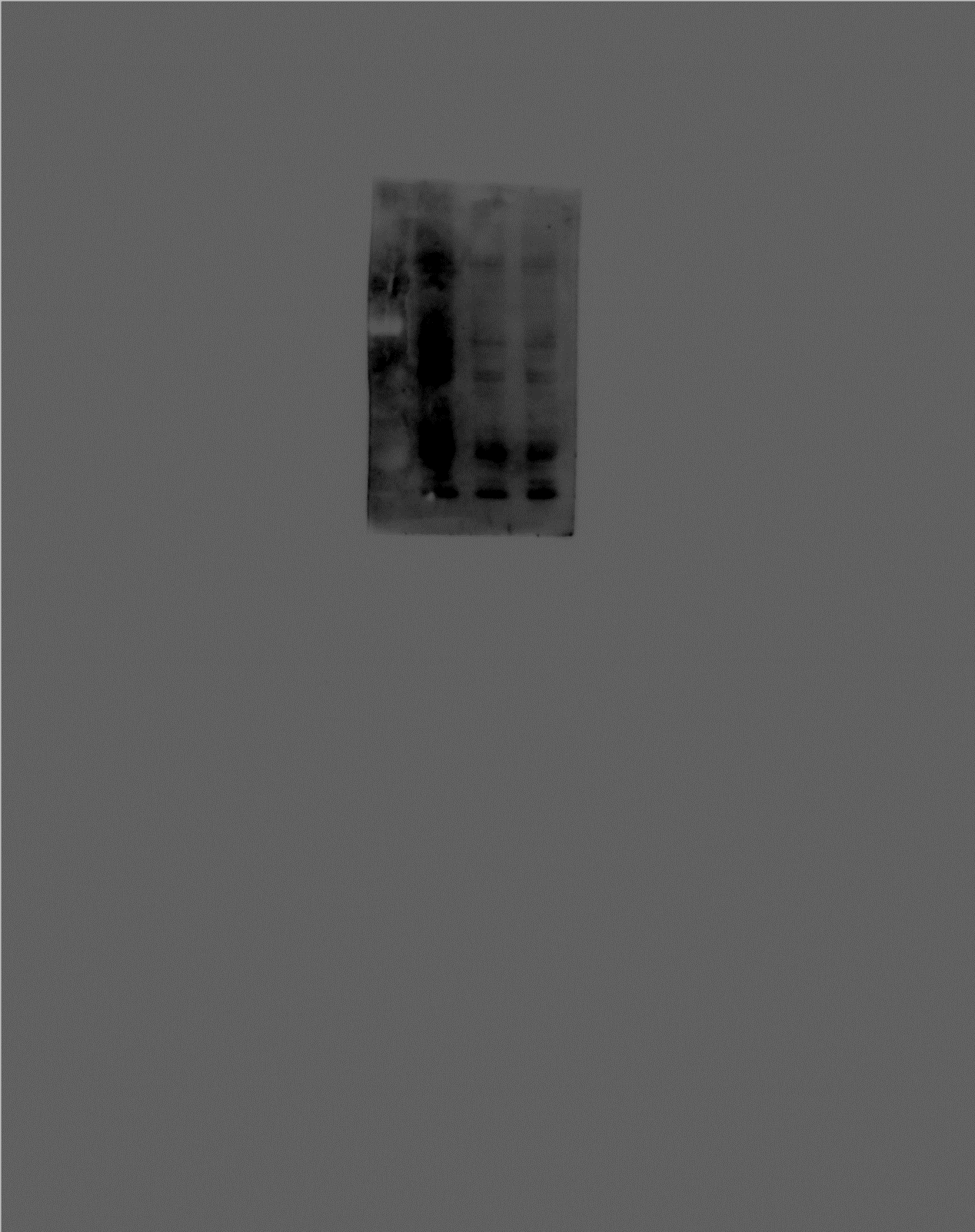 | 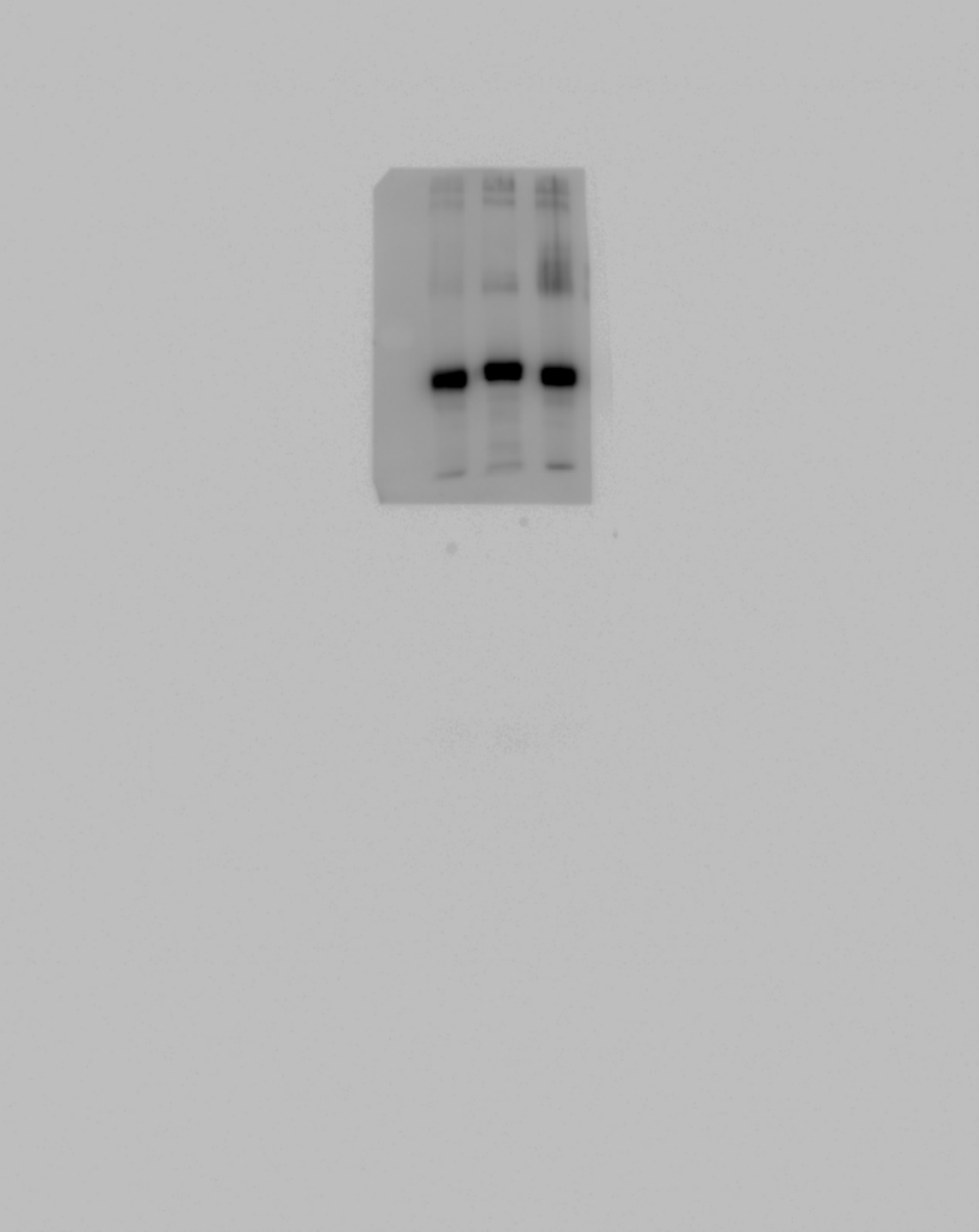 |
| β-actin（Lane 4-5-6） | NEMO（Lane 1-2-3） |
| 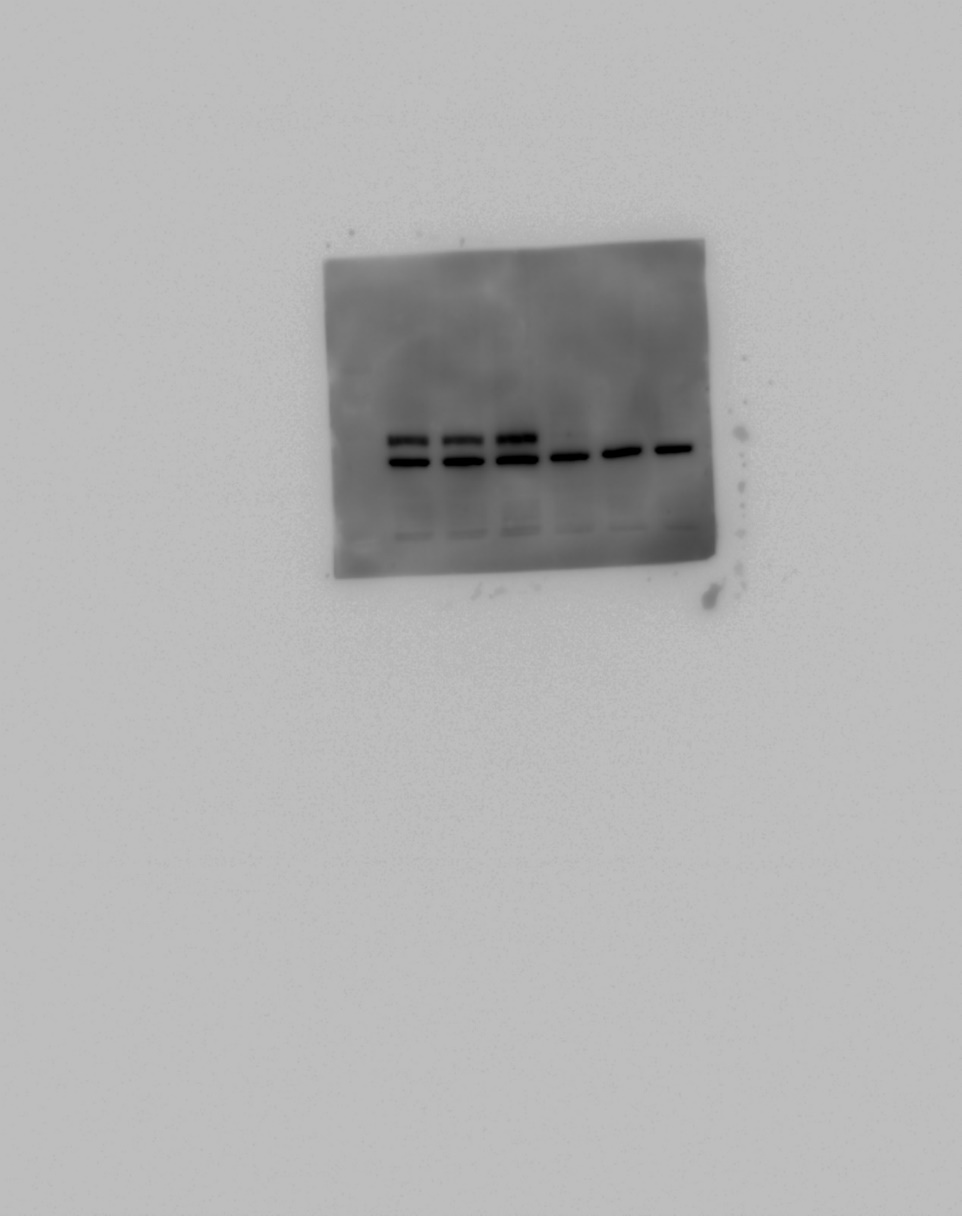 | 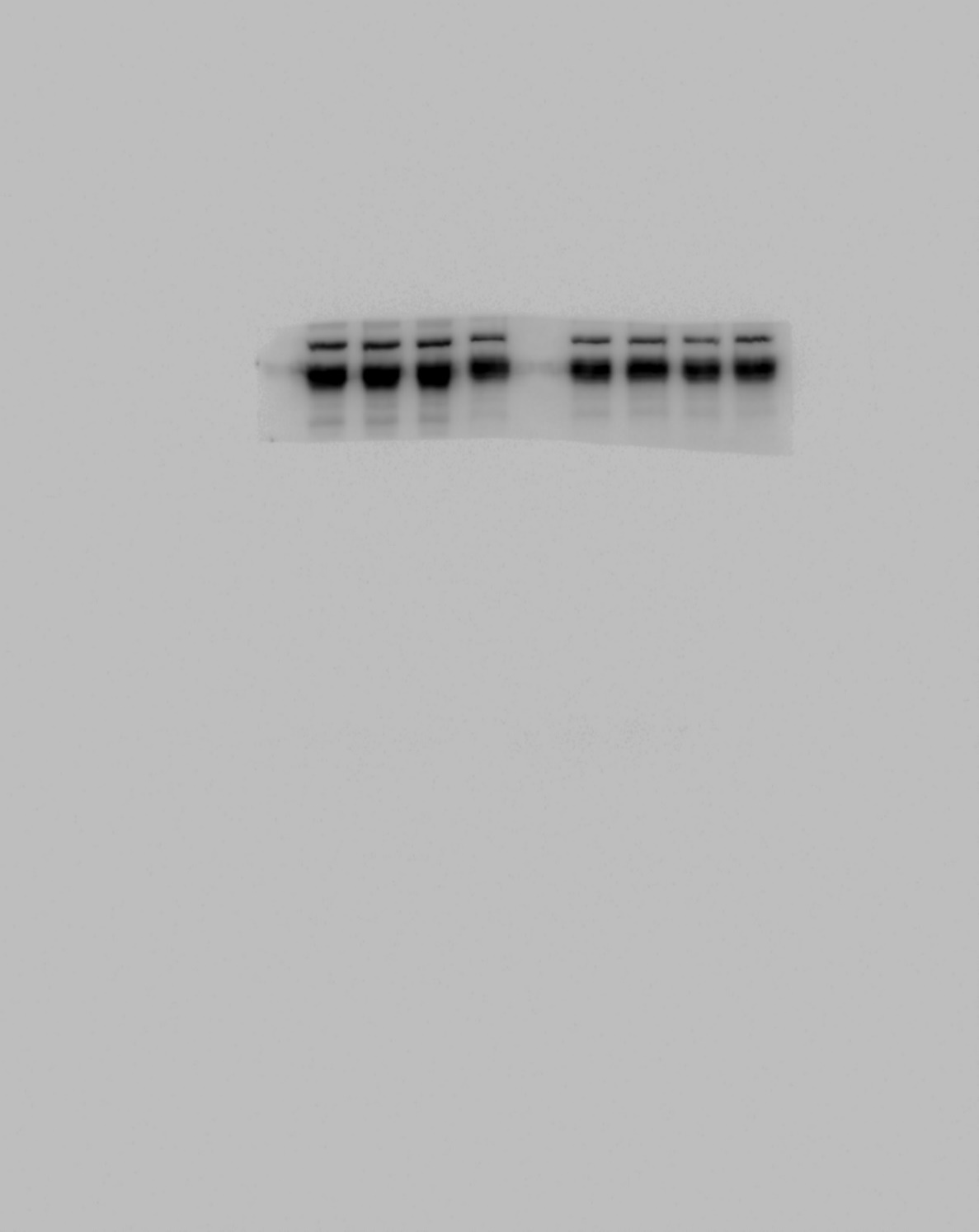 |

**Fig S-5: Original Western images used for preparing Figure 3E.**

| IκBα  （Lane 1-2-3） | 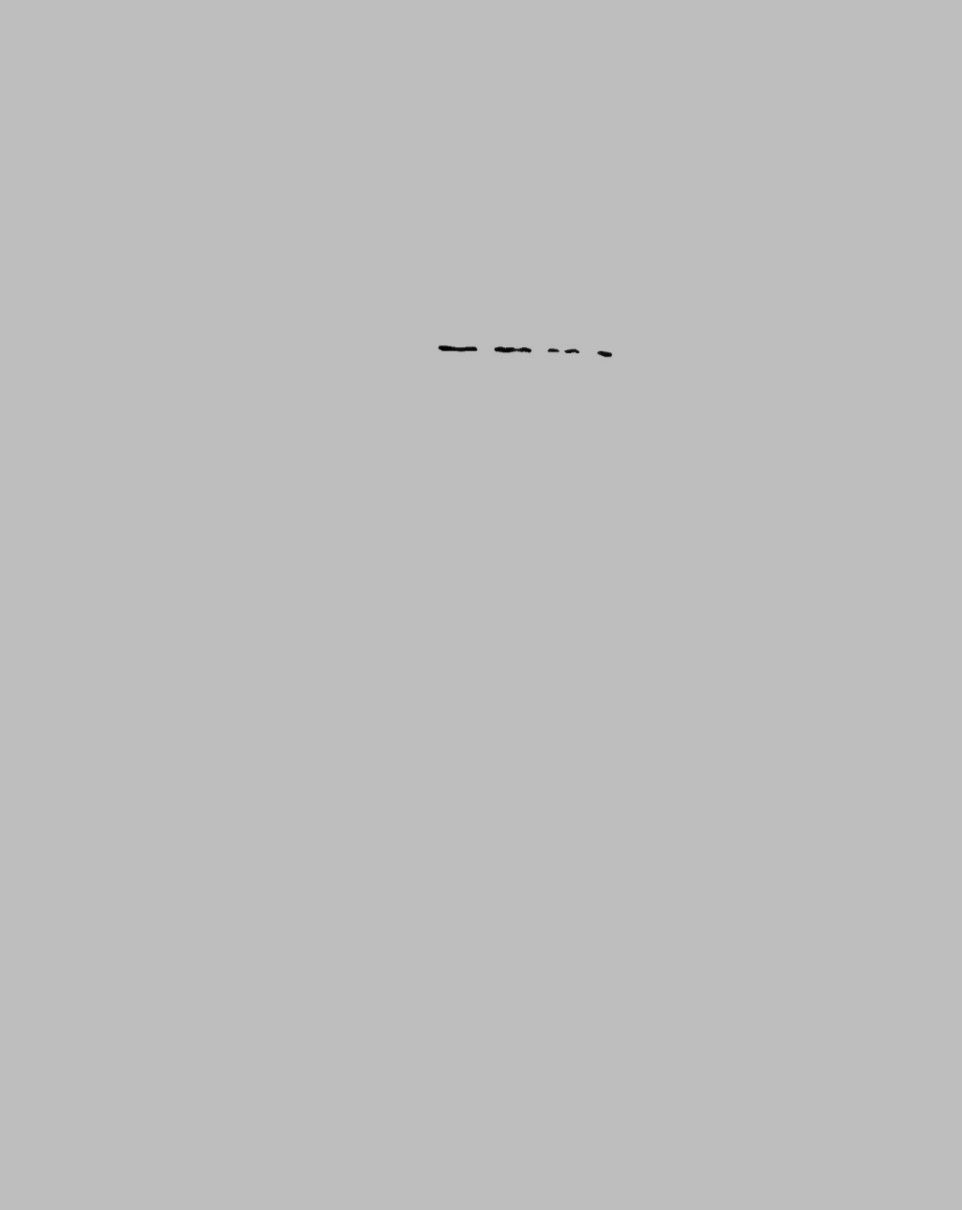 |
| --- | --- |
| p-P65  （Lane 2-3-4） | 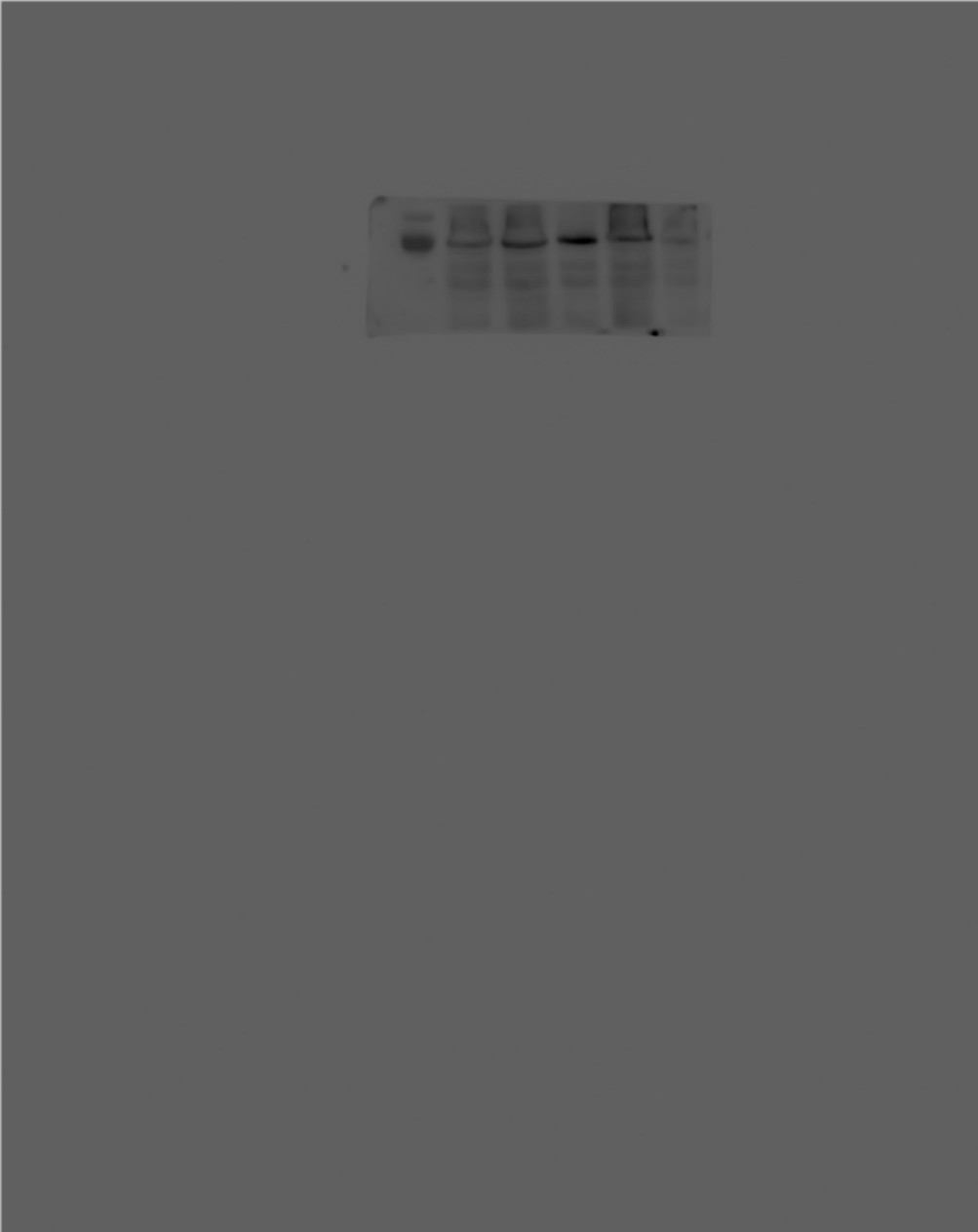 |
| β-actin  （Lane5-6-7） | 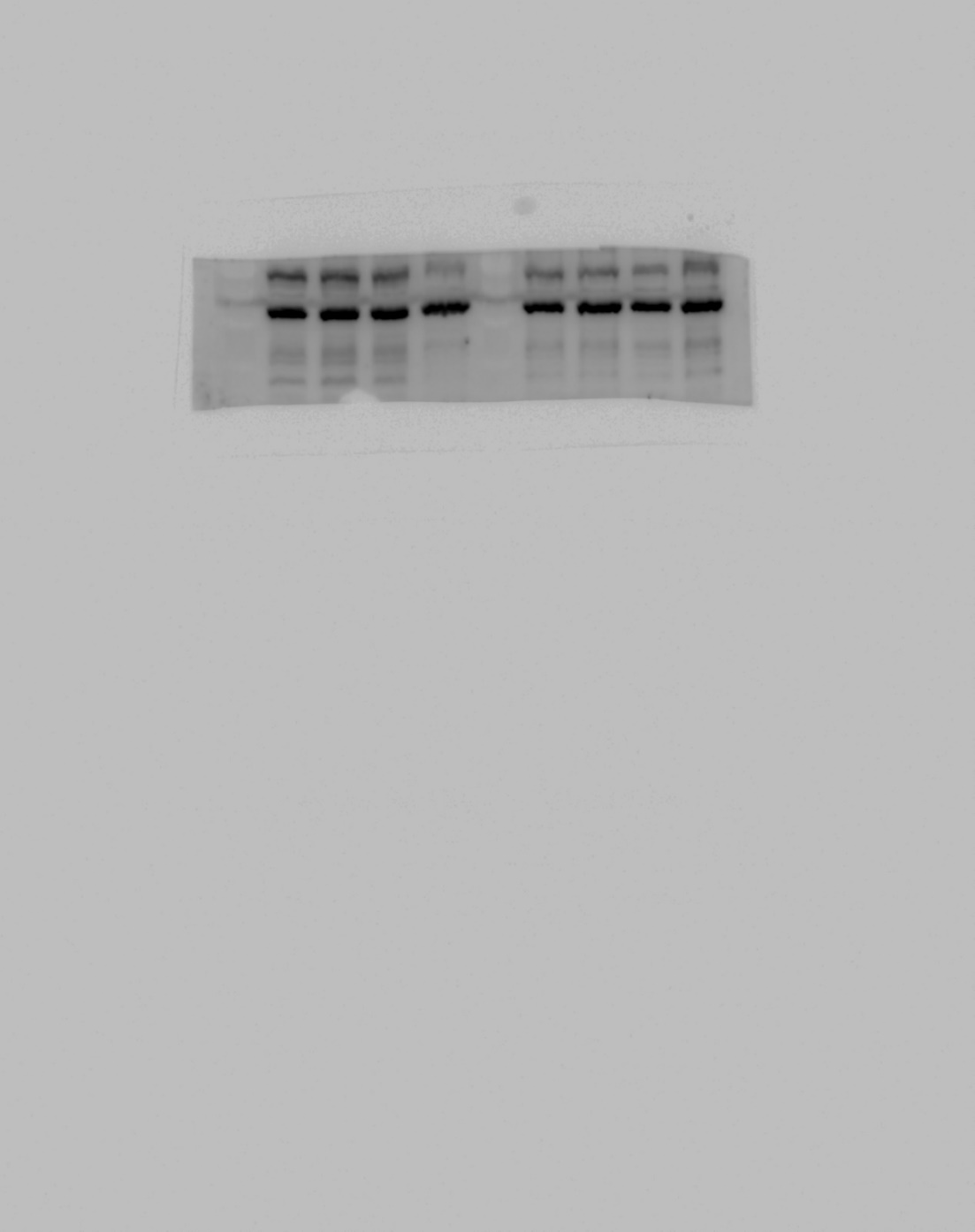 |

**Fig S-6: Original Western images used for preparing Figure 3F.**

| IκBα  （Lane 1-2-3）  The second row | 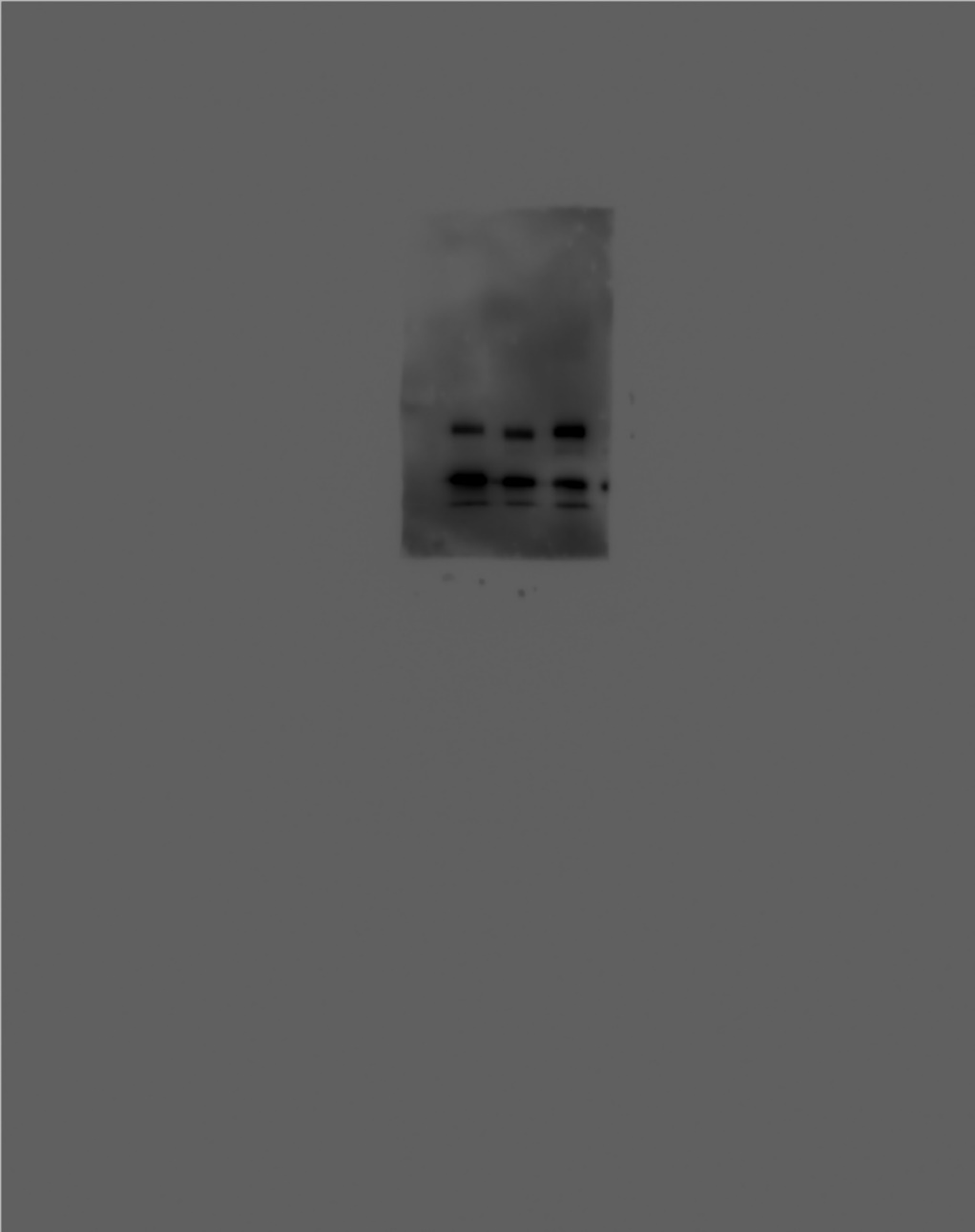 |
| --- | --- |
| p-P65  （Lane 2-3-4） | 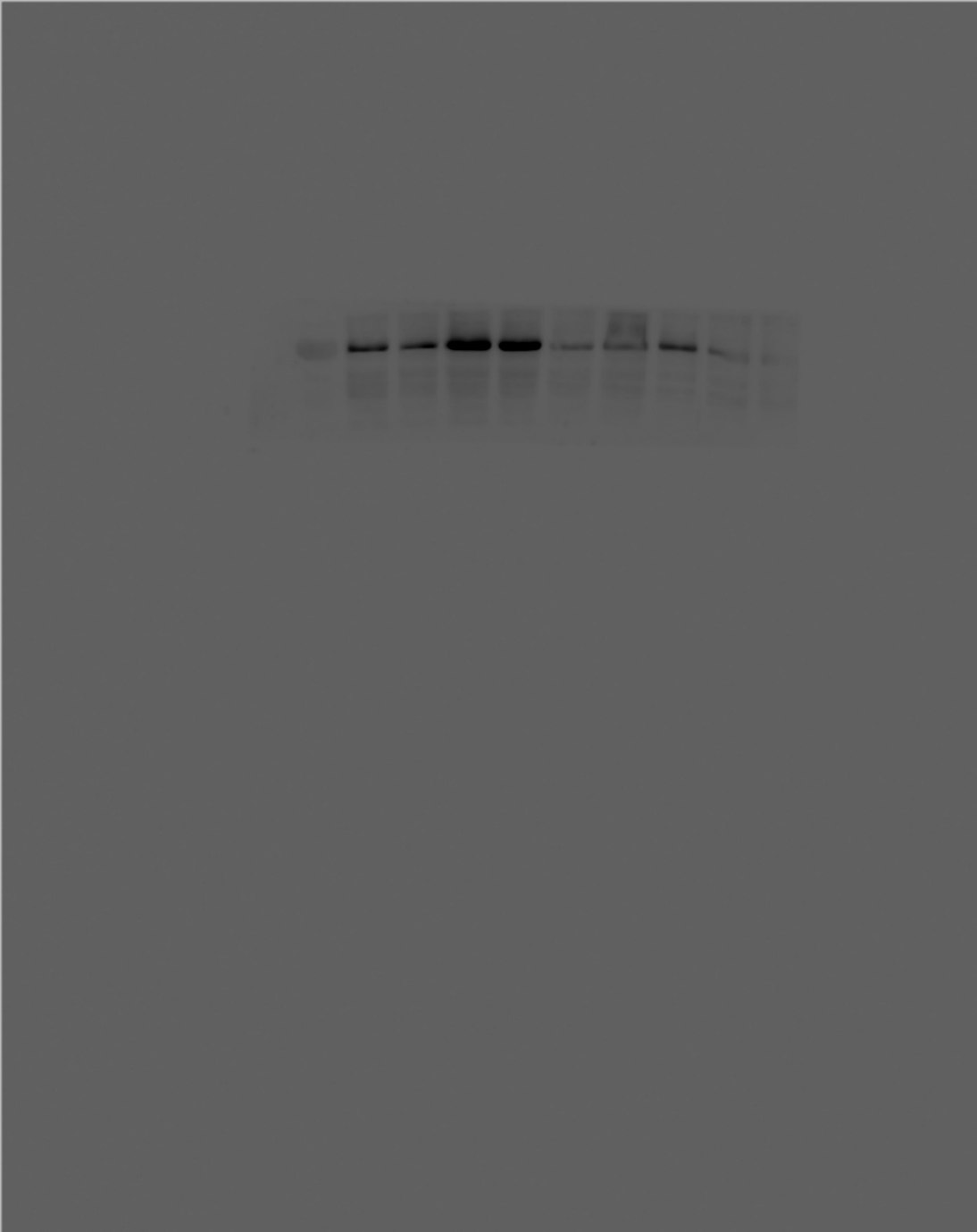 |
| β-actin  （Lane 5-6-7） | 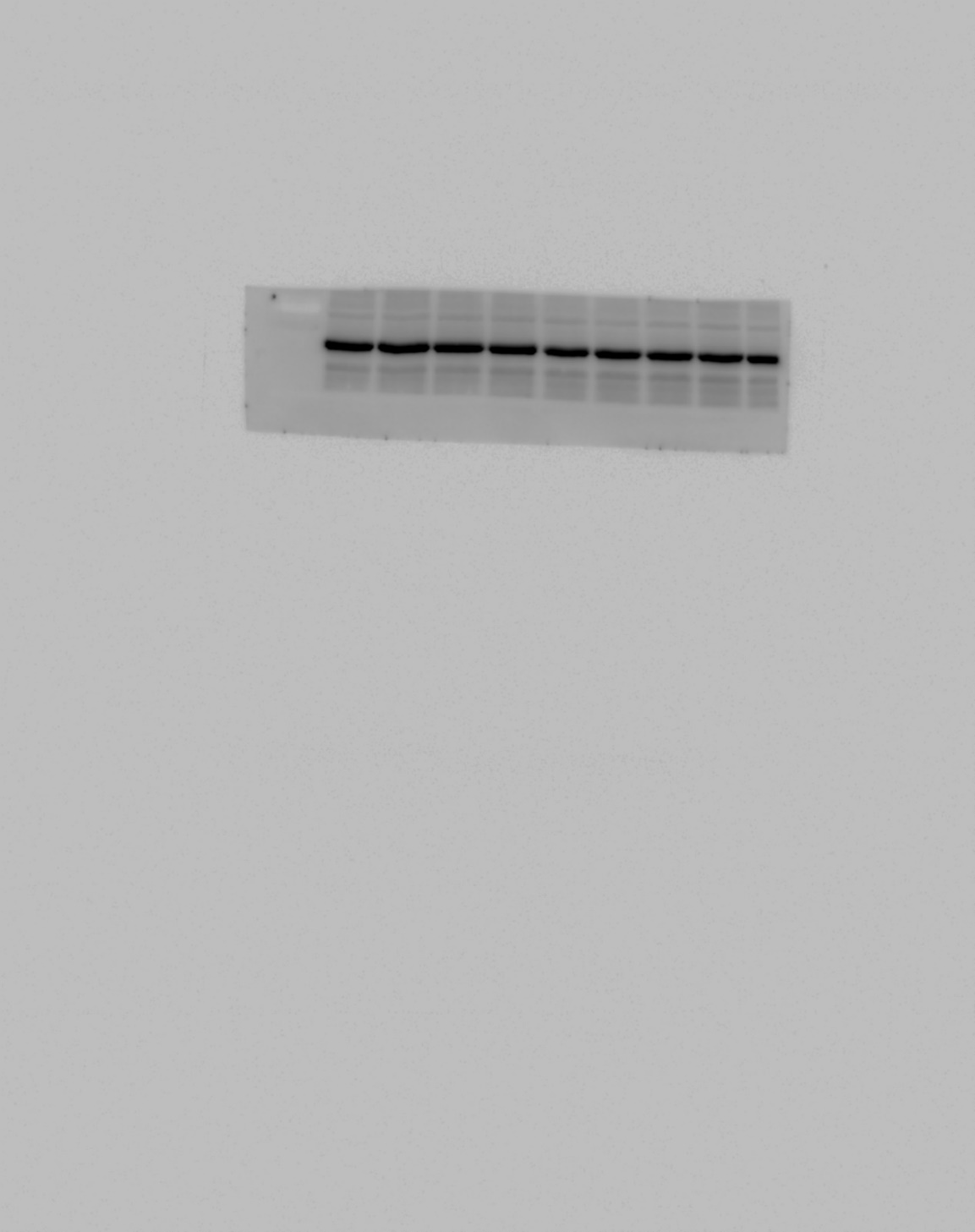 |
